# Glutamine alleviates immunosuppression in polymicrobial sepsis by augmenting bacterial phagocytosis through sustaining the GFAT-DRP1 dependent mitochondrial calcium dynamics

**DOI:** 10.1042/CS20256651

**Published:** 2025-10-22

**Authors:** Yuanfeng Zhu, Xiaoli Chen, Lin Xia, Shijun Fan, Qian Chen, Yan Wei, Yongling Lu, Xin Liu, Xi Peng

**Affiliations:** 1 Clinical Medical Research Center, Southwest Hospital, Third Military Medical University (Army Medical University), Chongqing, 400038, China; 2 State Key Laboratory of Trauma and Chemical Poisoning, Southwest Hospital, Third Military Medical University (Army Medical University), Chongqing, 400038, China

**Keywords:** DRP1, GFAT, glutamine, immunosuppression, mitochondrial, phagocytosis, sepsis

## Abstract

Sepsis triggers impaired macrophage bacterial phagocytosis, rendering the host more vulnerable to secondary infections, a manifestation termed sepsis-associated immunosuppression. Glutamine (Gln) is a vital nutrient in critical illness that not only supports energy production and biomass synthesis but also potentially exerts immunomodulatory effects. The aim of the present study was to investigate whether supplementation of Gln modulates macrophage phagocytosis and mitigates sepsis-induced immunosuppression. Using a murine model of polymicrobial sepsis, we evaluated the effects of Gln supplementation on bacterial load, cytokine production, and survival. In multiple *in vitro* assays, we employed molecular and pharmacological approaches to dissect Gln-dependent signaling pathways in recovering the immunosuppressive macrophages. We found that Gln deficiency impaired macrophage phagocytosis and exacerbated sepsis-induced immunosuppression. In contrast, exogenous Gln supplementation restored macrophage function and improved survival in septic mice—effects that were abolished upon macrophage depletion. Mechanistically, Gln promoted glutamine-fructose-6-phosphate transaminase (GFAT)-dependent protein O-GlcNAcylation, leading to dynamin-related protein 1 (DRP1) oligomerization. Concurrently, Gln activated a GFAT-mediated, cyclin-dependent kinase 1-dependent pathway that induced DRP1 phosphorylation at Ser-616 irrelevant of O-GlcNAcylation. These effects enhanced DRP1-mediated mitochondrial fission, increased mitochondrial calcium efflux, and sustained cytosolic calcium levels essential for phagocytosis. In conclusion, our study demonstrates that Gln strengthens macrophage phagocytosis and alleviates immunosuppression in sepsis through a dual GFAT-DRP1 mechanism co-ordinating mitochondrial dynamics and calcium signaling, highlighting the GFAT–DRP1–calcium axis as a potential therapeutic target for treating sepsis-induced immunosuppression.

## Introduction

Sepsis is a life-threatening medical condition that arises when the body’s response to microbial infection becomes dysregulated. It primarily involves a complex interplay between intense hyperinflammation and persistent immunosuppression [1]. Advances in immune phenotypic studies have highlighted that sepsis-induced immunoparalysis impairs the ability to clear pathogens, thus rendering the host more vulnerable to secondary infections [2,3]. Phagocytosis is a conserved immune response essential for the timely recognition and elimination of pathogens or cell debris [4]. Notably, immunocompromised patients frequently exhibit impaired bacterial phagocytosis and clearance [4–6], a dysfunction closely related to the host’s inability to effectively eliminate invading pathogens during sepsis.

Macrophages are sentinel phagocytes responsible for detecting and eliminating invading microbes through processes such as phagocytosis, pathogen degradation, and antigen presentation. Compelling evidence indicates that macrophage phagocytosis is significantly impaired during the late stages of sepsis, leading to delayed pathogen elimination and contributing to the overall immunosuppressive state observed in patients and animal models [4,5,7,8]. Furthermore, enhancing phagocytotic capacity in macrophages may restore antimicrobial responses and mitigate sepsis-induced organ damage [5,9]. These findings underscore the scientific and translational importance of modulating macrophage phagocytosis in the context of sepsis. The process of phagocytosis begins with binding to phagocytic receptors, which triggers extensive actin polymerization to facilitate the capture and engulfing of pathogens [10]. Notably, the regulation of actin remodeling is calcium-dependent. In fact, calcium plays a direct role in dissolving the actin coat around the phagosome during particle uptake and is involved in recruiting calcium-dependent kinases such as protein kinase C, calmodulin, and annexin to phagosomes before further regulating subsequent events such as fusion and actin shedding [11]. A deeper understanding of the mechanisms governing calcium dynamics and calcium-dependent signals in macrophage phagocytosis is thus essential for developing novel antimicrobial strategies in sepsis.

Mitochondria support phagocytosis by fueling this energy-demanding process through ATP production. Defective bacterial phagocytosis has been associated with mitochondrial dysfunction across various pathological conditions, including chronic obstructive pulmonary disease [12], subarachnoid hemorrhage [13], and Alzheimer’s disease [14]. Additionally, recent studies have shown that macrophage phagocytosis of both tumor cells and apoptotic cells can be enhanced through mitochondrial fission—a process driven by dynamin-related protein 1 (DRP1) that facilitates the removal of damaged mitochondria under stress conditions [15,16]. Intriguingly, DRP1-dependent fission also regulates mitochondrial calcium dynamics by promoting mitochondrial calcium efflux [17]. This not only prevents mitochondrial calcium overload and preserves mitochondrial integrity but also increases cytosolic calcium levels to potentiate phagocytosis [15,18]. Despite these advances, the underlying mechanisms governing mitochondrial fission and calcium dynamics—and thereby affecting sepsis-associated immunosuppression—remain unclear.

Growing evidence suggests that nutrient availability in the microenvironment significantly influences immune cell metabolism and function, including macrophage phagocytosis [19]. Glutamine (Gln) is a key nutrient supporting the energy demand and synthetic metabolism in critical illness while also known for its immunomodulatory, anti-inflammatory, and antioxidative properties [20–22]. The decline of plasma Gln, as commonly observed in critically ill patients, is associated with injury-induced immune dysfunction following major burn injury [23]. Importantly, effective macrophage phagocytosis also depends on sufficient Gln availability, as demonstrated in tumor and neurodegenerative contexts [24–26]. However, it remains unclear whether Gln deficiency contributes to impaired phagocytosis and immunosuppression in sepsis. Herein, we hypothesize that Gln enhances macrophage phagocytosis and alleviates immunosuppression in sepsis by promoting DRP1-mediated mitochondrial fission and regulating calcium dynamics. To test this, we assessed the ability of Gln to enhance bacterial phagocytosis and reverse immunosuppression in a murine model of polymicrobial sepsis. We further investigated whether these effects depend on glutamine-fructose-6-phosphate transaminase (GFAT)-driven activation of DRP1, mitochondrial fission, and subsequent calcium mobilization. Our findings aim to reveal a novel signaling axis through which Gln availability recovers macrophage immune function and highlight its therapeutic potential in treating sepsis-induced immunosuppression.

## Methods

### Chemicals and reagents

Dulbecco’s Modified Eagle’s Medium (DMEM) with and without L-Gln (C11995500BT, 11960044), DMEM with and without calcium ions (C11995500BT, 21068028), pHrodo™ deep red *Escherichia coli* (*E. coli*) BioParticle (P35361), pHrodo™ green *Staphylococcus aureus* (*S. aureus*) BioParticle (P35382), Fluo-4, AM (F14201), Rhod-2, AM (R1245MP), and disuccinimidyl suberate (DSS, A39267) were purchased from Thermo Fisher Scientific (Waltham, MA, U.S.A.). J774A.1 (TIB-67) and RAW264.7 (TIB-71) macrophage cell lines were purchased from ATCC (Rockefeller, MD, U.S.A.). Fetal bovine serum (FBS, ST30-3302P) was purchased from PAN-Biotech (Aidenbach, Germany). 6-Diazo-5-oxo-L-nor-leucine (DON, HY-108357), CB-839 (HY-12248), Mdivi-1 (HY-15886), 5-Aminoimidazole-4-carboxamide-1-β-D-ribofuranoside (AICAR, HY-13417), (R)-N-(furan-2-ylmethyl)-2-(2-methoxyphenyl)-2-(2-oxo-1, 2-dihydroquinoline-6-sulfonamido)-N-(thiophen-2-ylmethyl)acetamide (OSMI-1, HY-119738), Thiamet G (HY-12588), Avotaciclib hydrochloride (HY-137432B), O-(2-acetamido-2-deoxy-D-glucopyranosylidene) amino N-phenyl carbamate (PUGNAc, HY-108241), (1Z, 1’Z)-N’, N’’-(5, 5’-(thiobis(ethane-2, 1-diyl))bis(1, 3, 4-thiadiazole-5, 2-diyl))bis(2-phenylacetimidic acid) (BPTES, HY-12683), and CGP37157 (HY-15754) were purchased from MCE (Monmouth Junction, NJ, U.S.A.). L-Gln (49419), lipopolysaccharides (LPS, L9143), ethylene glycol-bis(2-aminoethylether)-N, N, N′, N′-tetraacetic acid (EGTA, E3889), GTP agarose (G9768), and fluorescent yellow-green latex beads (L1030) were purchased from Sigma Aldrich (St. Louis, MO, U.S.A.). BAPTA, AM was purchased from Selleck (S7534, Houston, TX, U.S.A.). MitoTracker was purchased from Solarbio Life Sciences (M9940, Beijing, China). Primary antibodies against DRP1 were purchased from Santa Cruz Biotechnology (sc-271583, Dallas, TX, U.S.A.) and Abcam (ab184247, Cambridge, U.K). Primary antibody against O-linked N-acetylglucosamine (O-GlcNAc) was purchased from PTM BIO (PTM-951RM, Zhejiang, China). Primary antibodies against p-DRP1 (Ser-616, ab314755), TOMM20 (ab186735), GFAT1 (ab125069), and GFAT2 (ab190966) were purchased from Abcam (Cambridge, U.K). Primary antibodies against AMPK (2532), p-AMPK (2535), CAMKK2 (16810), p-CAMKK2 (ab190966), P38 (9212), p-P38 (4511), ERK1/2 (4695), and p-ERK1/2 (5726). Primary antibody against p-DRP1 (Ser-637, PA5-37534) was purchased from Thermo Fisher Scientific (Waltham, MA, U.S.A.). Primary antibodies against cyclin-dependent kinase 1 (CDK1) (19532–1-AP), PINK1 (23274–1-AP), and GSK3β (82061–1-RR) were purchased from Proteintech (Wuhan, China). Primary antibody against p-CDK1 (Thr-161) was purchased from ABclonal (AP0324, Wuhan, China). F-actin tracker (C2203S), primary antibodies against β-actin (AF0003) and α-tubulin (AF2827), HRP-conjugated (A0208, A0216), FITC-conjugated (A0562), Cy3-conjugated (A0521) secondary antibodies, and bovine serum albumin (BSA, ST023) were purchased from Beyotime Biotechnology (Shanghai, China).

### Animals

Wildtype Bagg’s albino/c (BALB/c) mice (male, 7–8 weeks old) were purchased from Vital River Laboratory Animal Technology Co., Ltd. (Beijing, China). All mice were housed under standard specific pathogen-free conditions with free access to water and food. All animal experiments were performed at the Experimental Animal Center of the First Affiliated Hospital of the Army Medical University and were approved by the Institutional Animal Ethics Committee of the Army Medical University and complied with the national and institutional guidelines for animal care and use (No. AMUWEC20201492).

### Macrophage depletion *in vivo*

BALB/c mice were injected intravenously daily with either control liposomes (CP-005–005, Liposoma, Amsterdam, Netherlands) or clodronate liposomes (CP-005–005, Liposoma, Amsterdam, Netherlands) (50 mg/kg body weight each) for two consecutive days. Macrophage depletion was confirmed by immunofluorescence detection of F4/80 (53–4801-82, Invitrogen, San Diego, CA, U.S.A.) in liver tissue and flow cytometry analysis of F4/80-positive cells in peritoneal fluids.

### Animal experiments

A sepsis-induced immunosuppression model was established in mice through secondary bacterial infection following cecum ligation and puncture (CLP). Briefly, mice were anesthetized by an isoflurane inhalation combined with sodium pentobarbital (40 mg/kg) and subjected to a single puncture with a 16-gauge needle 1 cm from the cecum tip. Subsequently, 24 h post-CLP surgery, *Pseudomonas aeruginosa* [*P. aeruginosa,* 1 × 10⁹ colony-forming units (CFU)/kg] was injected intravenously. Gln (0.1 g/kg body weight), alone or combined with DON (1 mg/kg body weight), was administered intravenously every 24 h after bacterial infection. The seven-day survival rate of the mice was observed. For further experimental analysis, CLP mice were killed by cervical dislocation 48 h after *P. aeruginosa* infection, and blood samples were collected for bacterial load quantification and enzyme-linked immunosorbent assay (ELISA). Additionally, peritoneal macrophages were harvested for the detection of intracellular calcium ions, mitochondrial morphology, and Western blot analysis. After the experiments were completed, all the surviving mice were killed by inhalation of excessive carbon dioxide.

### Bacterial culture

Standard strains of *E. coli* (35218)*, S. aureus* (25923)*,* and *P. aeruginosa* (27853) were purchased from ATCC (Manassas, VA, U.S.A.). Inoculate the bacterial strains into lysogeny broth medium, culture at 37℃ until logarithmic phase (OD600=0.6–0.8), and then dilute to achieve a multiplicity of infection (MOI) of one per cell before further use.

### Isolation and induced differentiation of bone marrow-derived macrophages

The mice were killed using the cervical dislocation method and soaked in 75% ethanol. The hind legs’ femur and tibia were aseptically obtained. The muscles and connective tissue were removed, and both ends of the femur and tibia were cut off, exposing the bone marrow cavity. The bone marrow cavity was rinsed three times with phosphate-buffered saline (PBS), and bone marrow fluid was collected. After centrifugation, the red blood cells were lysed, and the remaining cells were seeded in DMEM (containing 10% FBS and 10% L929 culture supernatant) and cultured at 37℃ in a humidified incubator supplemented with CO2 (5%). The cells differentiate into macrophages after 5–7 days.

### *In vitro* endotoxin tolerance model

Macrophages were pretreated with LPS (10 ng/ml) for 24 h to induce the immunosuppression model, followed by treatment with LPS (100 ng/ml) or other reagents for subsequent experiments and detection.

### Mitochondria isolation

Mitochondrial isolation was performed using a mitochondrial isolation kit (HY-K1060, MCE, Monmouth Junction, NJ). In brief, cells (2 × 10^7^) were lysed with a mitochondrial separation reagent (1 ml) in an ice bath for 15 minutes (min). The cells were well homogenized, and then the homogenate was centrifuged at 4°C, 600 ***g*** for 10 min. The supernatant was transferred to a clean eppendorf (EP) tube and centrifuged at 4°C, 11,000 ***g*** for 10 min. The precipitate was purified cellular mitochondria. The mitochondria were lysed with a mitochondria lysis buffer for 20 min, and the mitochondrial proteins were further detected by Western blot.

### Phagocytosis assay

Phagocytosis was measured by a CFU assay or fluorescent assay, as we described previously [27]. Firstly, the cells (5 × 10^5^) were infected with *E. coli* and *S. aureus* for 2 h. Extracellular bacteria were killed by supplementation with 100 µg/ml ceftazidime. The cells were washed three times with sterile PBS to remove any residual antibiotic and lysed with saponin (0.3%, 47036–50G-F, Sigma Aldrich). The lysate was diluted, added to a nutrient agar plate, and incubated for 12 h at 37°C. The CFUs were detected using a SHINESO colony counter (Hangzhou, China). Bacterial uptake was assessed by measuring the phagocytic index, which was calculated as the ratio of intracellular bacteria to the total number of added bacteria. Secondly, macrophages (2 × 10^5^) were seeded onto glass-bottom dishes. Subsequently, pHrodo™ deep red *E. coli* BioParticles (Red-*E. coli*) or pHrodo™ green *S. aureus* BioParticles (Green-*S. aureus*) were introduced and allowed to incubate at 37°C for 1 h. The cells were thoroughly washed with PBS, fixed with paraformaldehyde (4%), and counterstained with DAPI (C1005, Beyotime). The images were acquired using the Zeiss LSM880 laser scanning confocal microscopy (LSCM, Oberkochen, Germany). To quantify phagocytosis, the mean fluorescence intensity (MFI) of at least 100 cells was calculated.

### Cytosolic and mitochondrial calcium ion assays

Cytosolic and mitochondrial calcium ion levels were detected by Fluo-4, AM, and Rhod-2, AM, respectively. In brief, cells (5 × 10^5^) were seeded in glass-bottom dishes or 24-well plates and treated as indicated. Fluo-4, AM (2 μM) or Rhod-2, AM (2 μM) was added to the culture supernatant and incubated for 30 min. Calcium ion levels were detected by LSCM or ACEA Novocyte flow cytometry (San Diego, CA, U.S.A.).

### Mitochondrial morphological determination

Mitochondrial morphology was detected by LSCM and transmission electron microscopy (TEM). For the LSCM assay, macrophages (1 × 10^5^) were seeded in glass-bottom dishes, treated as indicated, and then the cells were incubated with MitoTracker (0.5 µM) at 37°C for 20 min. After being washed three times with PBS, the fluorescence images were detected by LSCM. For the TEM assay, macrophages (5 × 10^5^) were seeded in six-well plates and treated as indicated. Cells were fixed with glutaraldehyde and dehydrated with gradient concentrations of ethanol and then placed in embedding boards containing epoxy resin. After polymerization at 60°C for 48 h, the samples were cut into ultra-thin sections with a thickness of 70 nm and collected on naked copper-mesh grids. The grids were stained with uranyl acetate and lead citrate and examined by TEM (Hitachi HT-7800, Tokyo, Japan).

### Knockout of DRP1 in macrophages by CRISPR/Cas9

The Cas9 lentivirus containing specific guide RNA for DRP1 was designed and purchased from SIDBIO. J774A.1 cells were infected with lentivirus at a MOI of 20 for 72 h. DRP1-knockout cells for subsequent experiments were selected by exposure to puromycin (60209ES, 10 μg/ml, Yeasen, Shanghai, China).

### Overexpression of wildtype and O-GlcNAcylation site mutant DRP1 by lentiviral infection

Specific targeted macrophages overexpression lentivirus of DRP1 wildtype (DRP1^WT^) or glycosylation site mutations (threonine at sites 604 and 605 was mutated to alanine) DRP1 (DRP1^MU^) was designed and purchased from Gene (Shanghai, China). DRP1-knockout J774A.1 cells were infected with overexpression lentivirus at a multiplicity of infection of 20 for 72 h. DRP1-overexpression cells for subsequent experiments were selected by exposure to puromycin (10 μg/ml).

### GFAT activity assay by LC/MS

The activity of GFAT is measured by the amount of final product UDP-GlcNAc [15]. The Orbitrap Exploris 120 Mass Spectrometer (Thermo Fisher Scientific) was used to analyze UDP-GlcNAc. The mass spectrometer was operated in negative ion mode and set to perform a complete FT-MS scan (m/z 70–1050, resolution=60,000). The sample was loaded into the Ultra High Performance Liquid Chromatography (UHPLC) system and separated by a Hydrophilic Interaction Liquid Chromatography (HILIC) column (ACQUITY UPLC BEH Amide, 130 Å, 1.7 µm × 2.1 mm × 100 mm, Waters, Milford, MA), which is directly coupled to the mass spectrometer. Solvent A consisted of ammonium acetate (20 mM) in Acetonitrile (ACN, 40%) with a pH of 9, while solvent B was pure ACN. These were used as mobile phases for UHPLC separation. The solvent gradient in UHPLC separation started from 60% to 99.9% solvent A at a flow rate of 400 µl/min within 0–3 minutes. The total chromatographic separation time for each analysis was 7 minutes. The fragmentation reactions of m/z 606.08 to 384.99 for UDP-GlcNAc were selected for quantitation using the chromatographic peak area in the selected ion chromatogram (SIC).

### GTP-binding activity assay

The GTP-binding activity assay was performed according to a previously described method [28]. Briefly, macrophages (5 × 10^5^) were seeded in six-well plates and treated as indicated. The cells were washed with PBS and suspended in GTP-binding buffer (20 mM Tris-HCl, 5 mM MgCl_2_, 150 mM NaCl, 0.1% Triton X-100, 0.025 mM PUGNAc, and 2 mM PMSF). Samples were sonicated for 15 s and centrifuged at 4°C, 1500 ***g*** for 15 min, and the protein concentration was determined using the BCA Protein Assay Kit (P0010, Beyotime). Proteins (300 µg) were incubated with GTP-agarose beads (100 µl) in a total volume of 500 µl of GTP-binding buffer overnight at 4°C. After washing three times with GTP-binding buffer, bound protein was eluted by adding SDS-PAGE buffer (50 µl, P0015, Beyotime) and incubated at 100°C for 5 min. Quantification of DRP1 pulled down by GTP-agarose beads was performed by Western blot.

### Intracellular chemical cross-linking with DSS

Intracellular chemical cross-linking with DSS was performed according to the manufacturer’s protocol. In brief, cells were treated as indicated and then washed with PBS (pH=8.5). The cells were incubated with PBS (pH=8.5) containing 1 mmol/l DSS at 4°C with rotation for 3 h. The reaction was quenched by adding Tris (50 mM, pH=7.5) and incubating for 15 min at 4°C. The samples were mixed with Non-Reducing Lane Marker Sample Buffer (5×) (Thermo Fisher Scientific) and heated at 100°C for 5 min. The protein oligomer was detected by Western blot.

### Immunofluorescence

Macrophages (1 × 10^5^) were seeded in glass-bottom dishes, treated as indicated, and then fixed with paraformaldehyde (4%). After blocking with PBS containing BSA (5%), the cells were incubated with primary antibodies (1:400 dilutions) at 4°C for 20 h. After being washed three times with PBS, the secondary antibody (1:400 dilutions) was added and incubated for 1 h at room temperature. The fluorescence images were captured by LSCM.

### siRNA interference

J774A.1 cells (2 × 10^5^) were seeded in six-well plates. Specific siRNAs for GFAT1, GFAT2, or CDK1 (SIDBIO, Chongqing, China) were diluted with Opti-MEM (120 µl, 31985070, Thermo Fisher Scientific, Waltham, MA, U.S.A.) to a final concentration of 50 nmol/L, mixed with HiPerFect transfection reagent (12 µl, 301704, Qiagen, Dusseldorf, Germany), and incubated at room temperature for 20 min. The mixture of siRNA and transfection reagent was added to the cultured macrophages for a 48 h incubation. The efficiency of siRNA interference was determined by Western blot.

### Real-time PCR assay

mRNA was extracted using an mRNA extraction kit (DP419, Tiangen, Beijing, China) and reverse transcribed into cDNA with a ReverTra Ace-α kit (FSQ-201, Toyobo, Osaka, Japan) according to the operating instructions. The cDNA templates were then mixed with iTaq Universal SYBR Green Supermix (1725124, Bio-Rad, Hercules, CA, U.S.A.) and primers for target genes (sequences listed in Supplementary Table S1). Quantitative real-time PCR was performed using a CFX96 Real-Time System (Bio-Rad).

### Immunoprecipitation

Protein A/G Dynabeads of 25 µl (88802, Thermo Fisher Scientific) were mixed with anti-DRP1 antibody (5 μg) and incubated at room temperature with rotation for 1 h. The Dynabeads were washed three times with PBS. Proteins (500 µg) were mixed with the antibody-Dynabeads mixture and incubated at 4°C with rotation overnight. The proteins bound to the Dynabeads were eluted with an elution buffer. A loading buffer was added and incubated at 100°C for 5 min, and then the proteins were separated by SDS-PAGE electrophoresis and transferred to a polyvinylidene fluoride (PVDF, ISEQ00010, Sigma) membrane. After blocking with QuickBlock Blocking Buffer (P0231, Beyotime) for 1 h, the membrane was incubated with anti-DRP1 antibody (1:100) or anti-O-GlcNAc antibody (1:1000) at 4°C overnight. The membrane was washed with Tris-buffered saline with Tween 20 (TBST) and incubated with an HRP-conjugated secondary antibody (1:2500) for 1 h. After washing with TBST, the protein bands were detected using Super ECL Plus Western Blotting Substrate (BG0001, BIOGROUND, Chongqing, China) and visualized using a VILBER Fusion FX Bio Imaging System (Greater Paris region, France).

### Western blot analysis

Macrophages were treated as indicated and then lysed with T-PER protein extraction reagent (78510, Thermo Fisher Scientific) containing a protease and phosphatase inhibitor mixture (04693159001, 4906837001, Roche, Mannheim, Germany). Proteins were separated by SDS-PAGE and transferred to PVDF membranes. After blocking with QuickBlock Blocking Buffer for 1 h, the membranes were incubated with the following antibodies at 4°C overnight: anti-DRP1 (1:100), anti-p-DRP1 (Ser-616) (1:1000), anti-p-DRP1 (Ser637) (1:1000), anti-O-GlcNAc (1:1000), anti-CAMKK2 (1:1000), anti-p-CAMKK2 (1:1000), anti-CDK1 (1:1000), anti-p-CDK1 (Thr161) (1:1000), anti-GFAT1 (1:1000), anti-GFAT2 (1:1000), anti-P38 (1:1000), anti-p-P38 (1:1000), anti-ERK1/2 (1:1000), anti-p-ERK1/2 (1:1000), anti-TOMM20 (1:1000), anti-PINK1 (1:1000), anti-GSK3β (1:1000), anti-β-actin (1:5000), anti-α-Tubulin (1:5000), and anti-GAPDH (1:2000). After washing with TBST, the membranes were incubated with HRP-conjugated secondary antibodies (1:2000) at room temperature for 1 h. The protein bands were detected using Super ECL Plus Western Blotting Substrate and visualized with a VILBER Fusion FX Bio Imaging system.

### Gln concentration assay in plasma

Supernatants from serum of CLP mice were collected at the indicated times. The Gln concentration was detected using a colorimetric method with a Gln detection kit (ab197011, Abcam).

### F-actin measurement

Macrophages (2 × 10^5^) were seeded in glass-bottom dishes and treated as indicated. The F-actin tracker was added and incubated for 20 min. F-actin levels were detected by LSCM and quantified as the MFI.

### ELISA

Supernatants of serum from CLP-induced immunosuppression mice were collected at the indicated time. TNF-α and IL-6 concentrations were measured by ELISA kits (88–7324-88, 88–7066-88, Thermo Fisher Scientific) according to the instructions.

### Statistical analysis

Quantitative data are expressed as mean ± standard deviation. The unpaired two-tailed student’s t-test was used for comparisons between two groups. Comparisons between multiple groups were made using one-way Analysis of Variance (ANOVA) with post hoc Bonferroni correction. *P*-values less than 0.05 were considered statistically significant.

## Results

### Gln alleviates sepsis-associated immunosuppression by enhancing macrophage bacterial phagocytosis

**Figure 1:**
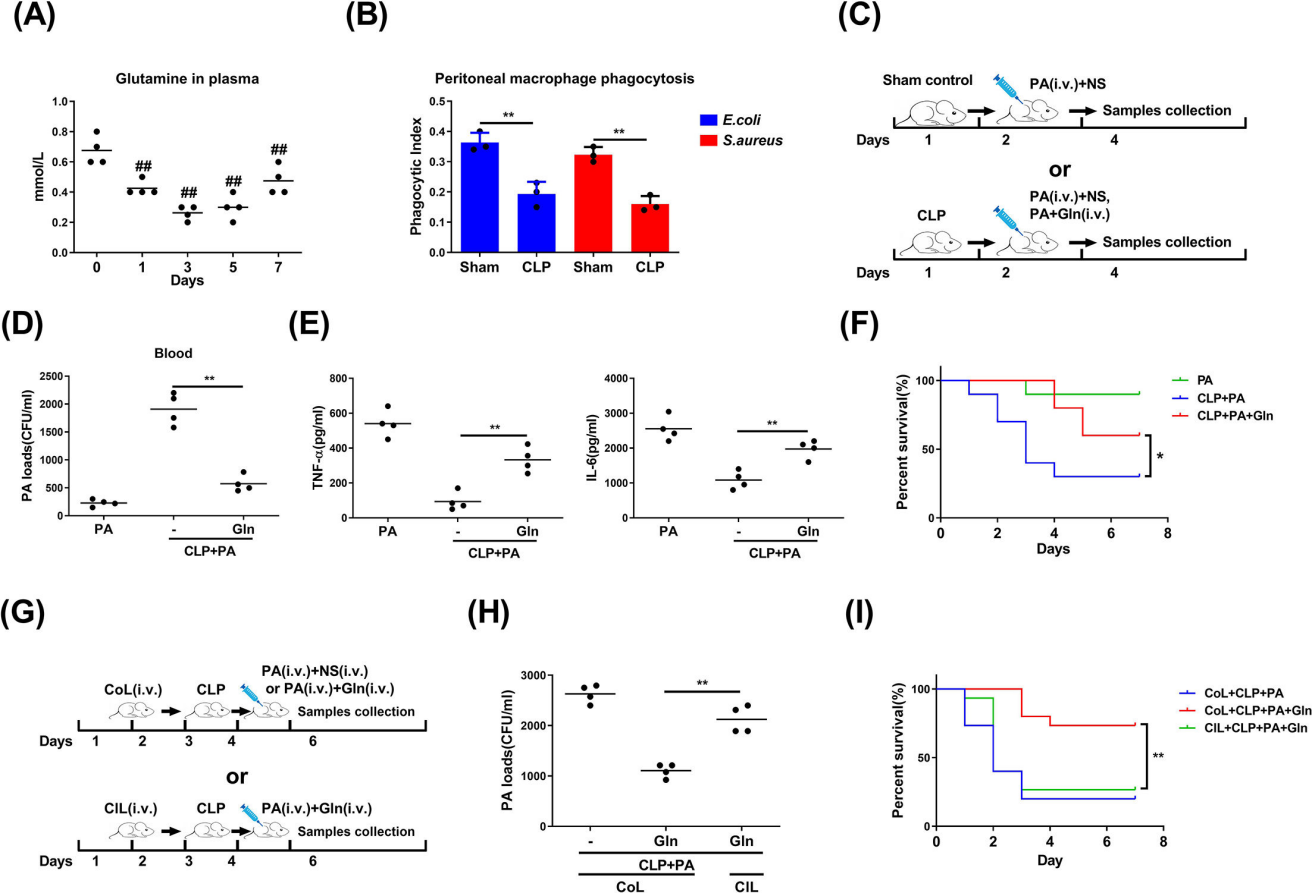
Glutamine alleviates sepsis-associated immunosuppression by enhancing macrophage bacterial phagocytosis. (**A**) Serum glutamine (Gln) concentration after CLP surgery was analyzed. (**B**) Phagocytosis of peritoneal macrophages in normal (Sham) and CLP mice 3 days after surgery was analyzed (*n*=3). (**C-F**) BALB/c mice were infected with *P. aeruginosa* (PA, 1 × 10^9^ CFU/kg body weight) 24 h after CLP surgery, and the mice were treated with normal saline (NS) or Gln (0.1 g/kg body weight) every 24 h (**C**). After 48 h, PA load (**D**), TNF-α, IL-6 (**E**) in serum and the survival rate of mice (**F**) (*n*=10) was observed. (**G-I**) Mice macrophages were depleted by injection of clodronate liposomes (ClL) or injection of control liposomes (CoL) as control. Then the mice were infected with PA (1 × 10^9^ CFU/kg body weight) 24 h after CLP surgery, and the mice were treated with Gln (0.1 g/kg body weight) every 24 h (**G**). After 48 h, PA load was analyzed (**H**), and seven day survival rate was observed (**I**) (*n*=10). ##: *P*<0.01 *vs* 0 day, **: *P*<0.01, *: *P*<0.05. *n*=4 for (**A**), (**B**), (**D**), (**E**), and (**H**).

Sepsis increased the demand and consumption of Gln, resulting in its rapid decline in circulation [23,29,30]. We initially addressed whether Gln availability was reduced in sepsis and compromised bacterial phagocytosis. Using a murine ‘two-hit model’ combining CLP with secondary infection of *P. aeruginosa*, we observed immunosuppressive manifestations, such as reduced TNF-α and IL-6 secretion, elevated bacterial load, and increased mortality upon secondary insult (Supplementary Figure S1A-D). Meanwhile, plasma Gln levels in CLP mice rapidly declined, reaching a minimum on the third day, concurrent with the onset of immunosuppression (Figure 1A). Phagocytic activity of peritoneal macrophages in CLP mice was also significantly lower than in sham mice, indicating the inability of CLP mice to eliminate secondary *P. aeruginosa* infection (Figure 1B). We then examined whether exogenous Gln supplementation could reverse sepsis-associated immunosuppression (Figure 1C). As expected, Gln administration improved outcomes in model mice subject to a secondary *P. aeruginosa* infection. Treatment led to a considerable reduction in bacterial load (Figure 1D), increased production of TNF-α and IL-6 (Figure 1E), and a significantly higher survival rate (Figure 1F). To confirm that Gln-mediated alleviation of immunosuppression in sepsis was linked with macrophage phagocytosis, clodronate liposomes were used to deplete macrophages and abolish macrophage-dependent phagocytotic capacity (Figure 1G and Supplementary Figure 2 Aand B). In Gln-treated immunosuppressed mice without macrophage phagocytosis, bacterial load dramatically increased (Figure 1H) and survival rate significantly decreased (Figure 1I). These findings suggest that Gln reverses immunosuppression in sepsis by selectively enhancing macrophage bacterial phagocytosis.

### Gln augments macrophage phagocytosis by increasing actin polymerization via elevating cytoplasmic calcium ion

**Figure 2:**
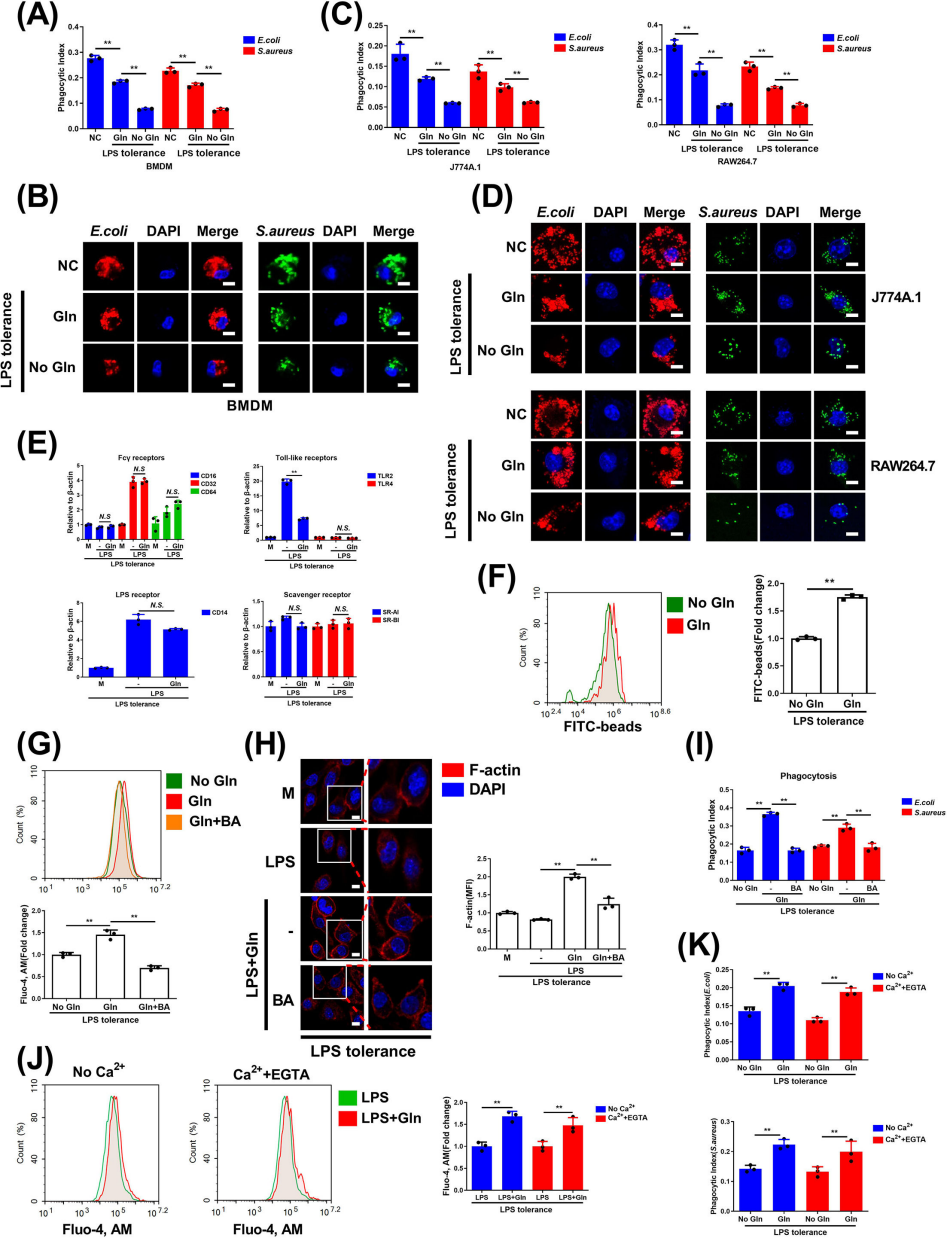
Gln augments macrophage phagocytosis by increasing actin polymerization via elevating cytoplasmic calcium ion (**A-D**) Normal (NC) or LPS-tolerant murine bone marrow-derived macrophages (BMDMs), J774A.1 and RAW264.7 cells were treated or untreated with Gln for 24 h, then infected with live *E. coli*, *S. aureus* (**A, C**), or pHrodo™ deep red *E. coli*, pHrodo™ green *S. aureus* (**B, D**) for 1 h. Phagocytosis was detected. (**E**) LPS-tolerant BMDMs were untreated or treated with LPS alone or with Gln for 24 h. The mRNA expression of phagocytic receptors [Fcγ receptors (CD16, CD32, and CD64), toll-like receptors (TLR2 and TLR4), LPS receptor (CD14) or scavenger receptor (SR-AI and SR-BI)] was detected. (**F**) LPS-tolerant BMDMs were untreated or treated with Gln for 24 h. Then added fluorescently labeled latex beads (FITC-beads) and incubated for 2 h. Phagocytosis was analyzed. (**G-H**) LPS-tolerant BMDMs were incubated in DMEM with or without Gln, or further treated with BAPTA, AM (BA) for 24 h. Cytosolic calcium ions (**G**) and fluorescence images of intracellular F-actin were detected (**H**). (**I**) LPS-tolerant BMDMs were incubated in DMEM with or without Gln, or further treated with BAPTA, AM for 24 h, and then infected with *E. coli* and *S. aureus* for 1 h. Phagocytosis was analyzed. (**J**) LPS-tolerant BMDMs were incubated in DMEM containing calcium ions plus EGTA or without calcium ions and further treated with LPS or together with Gln for 24 h. Cytosolic calcium ions were detected. (**K**) LPS-tolerant BMDMs were incubated in DMEM containing calcium ions plus EGTA or without calcium ions for 24 h, and then infected with *E. coli* and *S. aureus* for 1 h. Phagocytosis was analyzed. The concentration of LPS, Gln, EGTA, and BAPTA, AM was 100 ng/ml, 2 mM, 5 mM, and 10 μM respectively. The MOI for *E. coli* and *S. aureus* was 1. M: LPS-tolerant macrophages that have not been treated with Gln. **: *P*<0.01, *N.S*.: no significance. *n*=3. Scale bar = 5 μm.

Next, we conducted *in vitro* experiments using macrophages with LPS tolerance, mimicking the immunosuppressive state seen in sepsis (Supplementary Figure 3A-C). [31]. In this model, we examined the role of Gln in supporting macrophage phagocytosis. Live bacteria phagocytosis assays revealed that both primary macrophages (BMDMs) and macrophage cell lines (J774A.1 or RAW264.7) displayed significantly reduced ability to phagocytose live *E. coli* and *S. aureus* in tolerant macrophages (Figure 2A and C). The absence of Gln, induced by extracellular starvation, further impaired bacterial phagocytosis (Figure 2A and C). Confocal microscopy experiments confirmed these findings, showing decreased engulfment of fluorescently labeled *E. coli* and *S. aureus* in tolerant macrophages cultured without Gln (Figure 2B and D), underscoring the importance of Gln as a key nutrient in macrophage phagocytosis. To explore how Gln enhances macrophage phagocytosis, we examined whether Gln affects the expression of pathogen recognition receptors. Our results showed no increase in mRNA expression of receptors such as Fcγ receptors (CD16, CD32, and CD64), toll-like receptors (TLR2 and TLR4), LPS receptor (CD14) or scavenger receptor [scavenger receptor class A type I (SR-AI) and scavenger receptor class B type I (SR-BI)] in macrophages (Figure 2E). Yet, Gln enhanced the phagocytosis of fluorescent microspheres by LPS-tolerant macrophages (Figure 2F and Supplementary Figure 4A), indicating that phagocytic enhancement may be related to intracellular signaling rather than receptor modulation. Cytosolic calcium plays a crucial role in actin polymerization, a shared process in phagocytosis [11]. We thus investigated whether Gln affects calcium levels to promote phagocytosis. Calcium ion assays revealed that Gln significantly increased cytoplasmic calcium (Figure 2G and Supplementary Figure 4B) and actin polymerization (Figure 2H) in LPS-tolerant macrophages. This effect was blocked by BAPTA, AM, an intracellular calcium chelator, which reduced cytoplasmic calcium, actin polymerization, and Gln-induced phagocytosis (Figure 2G–I and Supplementary Figure 4C). Interestingly, neither calcium-free media nor extracellular calcium chelation with EGTA affected Gln-induced calcium increases or phagocytosis (Figure 2J and K and Supplementary Figure 4D), suggesting that Gln elevates cytoplasmic calcium independent of external calcium sources, likely through intracellular calcium pools. In summary, Gln enhances actin polymerization and macrophage phagocytosis by increasing cytoplasmic calcium levels, potentially involving intracellular calcium reservoirs.

### Gln facilitates mitochondrial calcium efflux by promoting DRP1-dependent mitochondrial fission in LPS-tolerant macrophages

**Figure 3:**
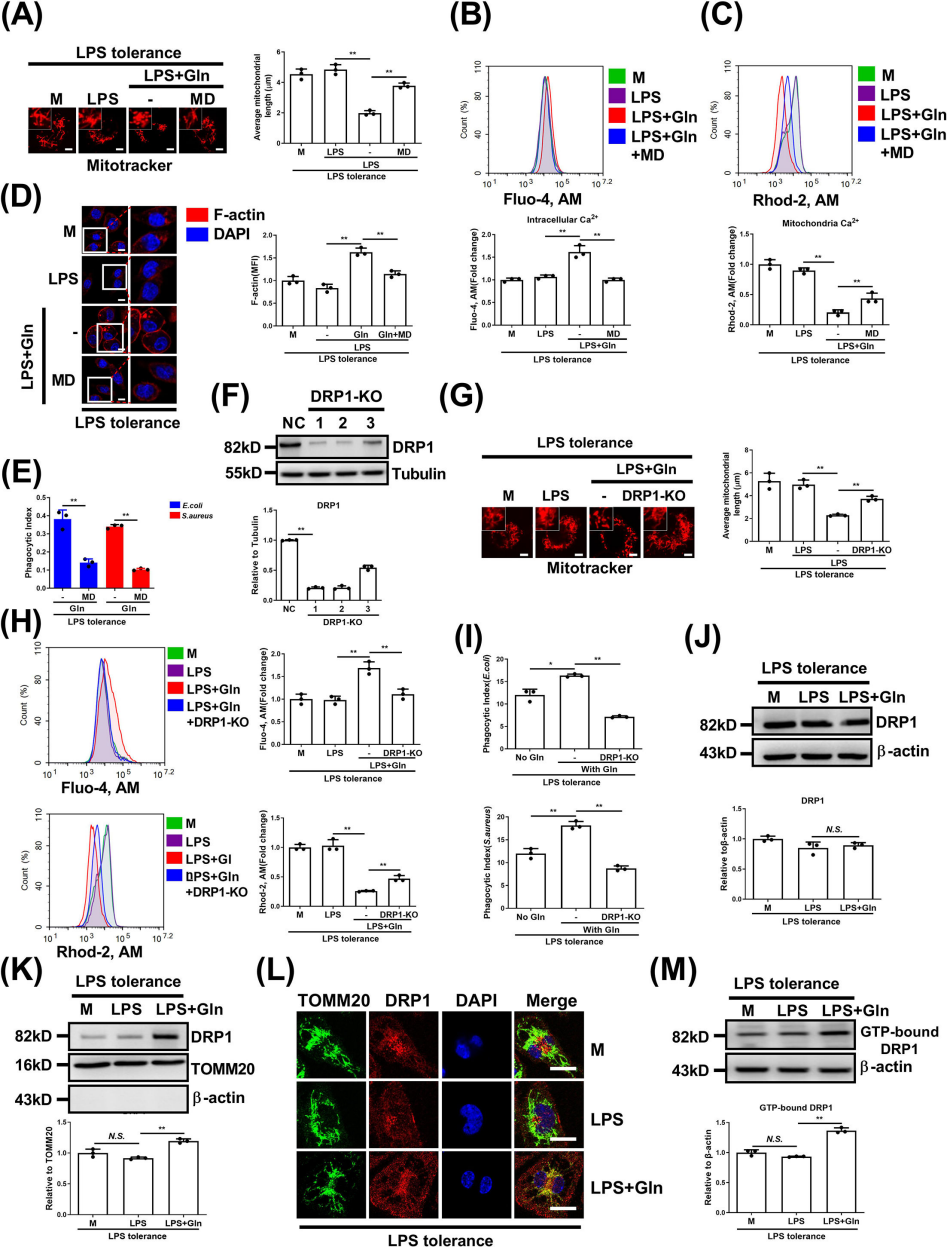
Gln facilitates mitochondrial calcium efflux by promoting DRP1-dependent mitochondrial fission in LPS-tolerant macrophages**.** (**A-D**) LPS-tolerant BMDMs were untreated or treated with LPS alone or together with Gln or further treated with Mdivi-1 (MD) for 24 h. The mitochondrial morphology (**A**) (Scale bar = 5 µm), Cytosolic calcium ions (**B**), mitochondrial calcium ions (**C**) and fluorescence images of intracellular F-actin (**D**) (Scale bar = 5 µm) were detected. (**E**) LPS-tolerant BMDMs were incubated in DMEM with or without Gln, or further treated with Mdivi-1 for 24 h, then infected with *E. coli* and *S. aureus* for 1 h. Phagocytosis was analyzed. (**F-H**) LPS-tolerant J774A.1 cells or DRP1 knockout (DRP1-KO) (**F**) LPS-tolerant J774A.1 cells were untreated or treated with LPS or together with Gln for 24 h. The mitochondrial morphology (**G**) (Scale bar = 5 µm), cytosolic calcium ions, and mitochondrial calcium ions (**H**) were detected. (**I**) LPS-tolerant J774A.1 cells or DRP1 DRP1-KO LPS-tolerant J774A.1 cells were incubated in DMEM with or without Gln for 24 h, then infected with *E. coli and S. aureus* for 1 h. Phagocytosis was analyzed. (**J-M**) LPS-tolerant J774A.1 cells were treated with LPS or together with Gln for 24 h. The protein expression of total (**J**), mitochondrial (**K**) DRP1, intracellular co-localization of DRP1 and TOMM20 (**L**) (Scale bar = 10 µm) and GTP-binding activity of DRP1 was detected (**M**). The concentration of LPS, Gln, and Mdivi-1 was 100 ng/ml, 2 mM, and 10 µM, respectively. The MOI for *E. coli* and *S. aureus* was 1. M: LPS-tolerant macrophages that have not been treated with Gln. **: *P*<0.01, *: *P*<0.05, *N.S*.: no significance. *n*=3.

Mitochondria serve as central metabolic hubs for buffering cytoplasmic calcium levels through DRP1-mediated fission [15,16]. We observed that Gln significantly promoted mitochondrial fission in LPS-tolerant macrophages, an effect suppressed by the fission inhibitor Mdivi-1 (Figure 3A and Supplementary Figure 5A). Mdivi-1 also inhibits the Gln-induced rise in cytoplasmic calcium (Figure 3B and Supplementary Figure 5B). In contrast with cytosolic calcium, mitochondrial calcium was significantly reduced by Gln but markedly elevated upon additional Mdivi-1 treatment (Figure 3C and Supplementary Figure 5C). Moreover, Mdivi-1 disrupted Gln’s ability to enhance actin polymerization (Figure 3D) and phagocytosis (Figure 3E and Supplementary Figure 5D) in LPS-tolerant macrophages. These results suggest that Gln promotes mitochondrial fission and calcium efflux, thus sustaining cytosolic calcium necessary for actin polymerization and phagocytosis in tolerant macrophages. Given that mitochondrial fission is mainly regulated by DRP1, with Mdivi-1 inhibiting this process, we hypothesized that Gln enhances DRP1 activity to induce fission and phagocytosis. To testify this hypothesis, we silenced DRP1 in LPS-tolerant macrophages (Figure 3F). Then DRP1 knockdown disrupted the elevation of macrophage mitochondrial fission induced by Gln (Figure 3G and Supplementary Figure 5E) while abolishing cytoplasmic calcium elevation and mitochondrial calcium reduction (Figure 3H and Supplementary Figure 6A). In line with this, Gln-induced macrophage phagocytosis was significantly reduced (Figure 3I and Supplementary Figure 6B). We further explored how Gln affects DRP1 activity. Although Gln did not increase DRP1 protein levels (Figure 3J), it significantly increased DRP1 mitochondrial localization (Figure 3K). The immunofluorescence assay confirmed greater DRP1 co-localization with the mitochondrial marker TOMM20 in Gln-treated macrophages (Figure 3L), indicating enhanced DRP1 translocation from the cytoplasm to mitochondria. Additionally, Gln significantly increased the GTPase activity of DRP1 (Figure 3M). In summary, Gln promotes mitochondrial fission and cytoplasmic calcium elevation by increasing DRP1’s mitochondrial translocation and activity in LPS-tolerant macrophages.

### Gln promotes DRP1 oligomerization through GFAT rather than via glutaminases

**Figure 4:**
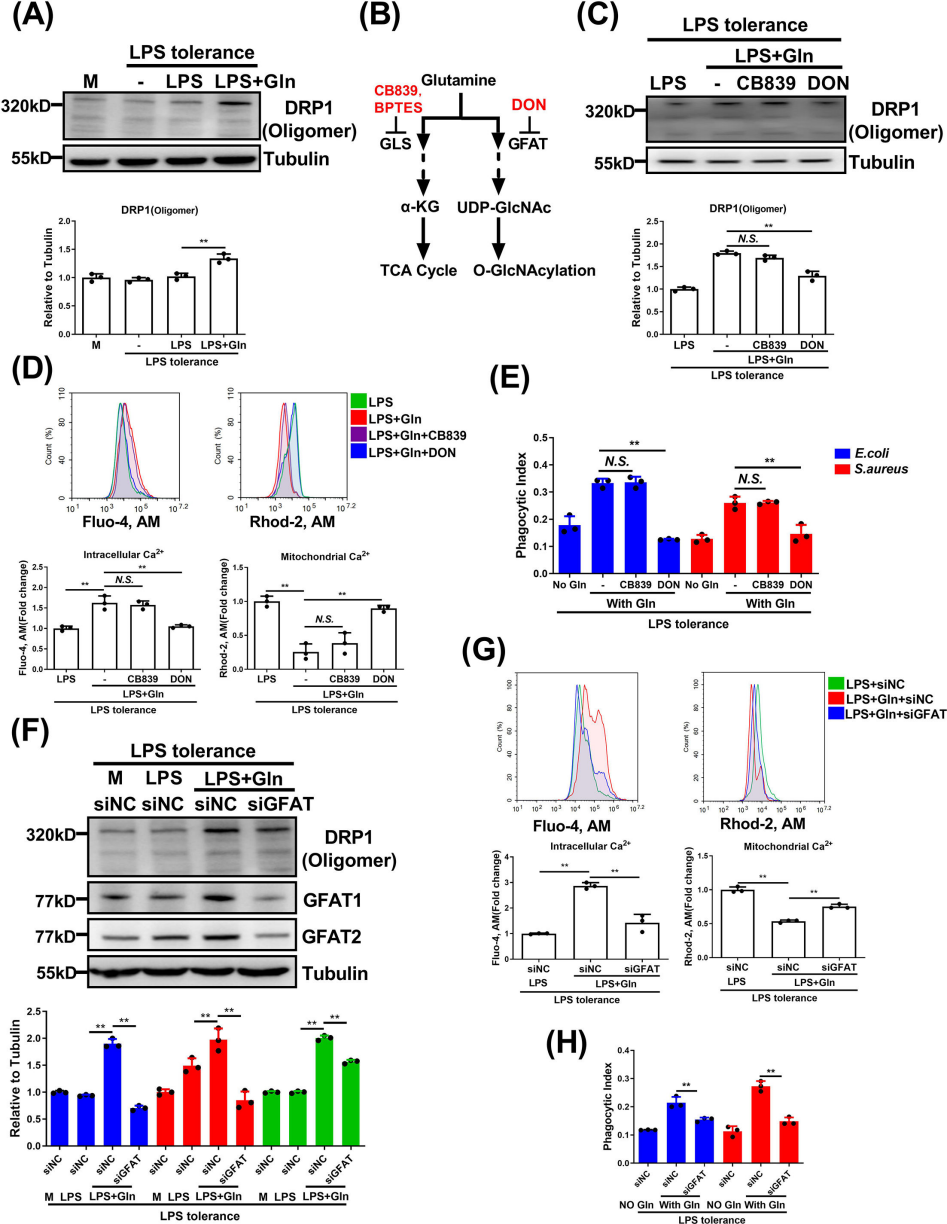
Gln promotes DRP1 oligomerization through GFAT rather than via GLSs. (**A**) Normal or LPS-tolerant BMDMs were untreated or treated with LPS or together with Gln for 24 h, then the cells were cross-linked for 3 h with DSS. DRP1 oligomer was detected. (**B**) The metabolic flowchart of Gln and the targets of inhibitors. (**C**) LPS-tolerant BMDMs were treated with LPS or together with Gln, or further treated with CB839 or DON for 24 h, then the cells were cross-linked for 3 h with DSS. DRP1 oligomer was detected. (**D**) LPS-tolerant BMDMs were treated as in (**C**). Cytosolic calcium ions and mitochondrial calcium ions were detected by flow cytometry. (**E**) LPS-tolerant BMDMs were incubated in DMEM with or without Gln, or further treated with CB839 or DON for 24 h, then infected with *E. coli* and *S. aureus* for 1 h. Phagocytosis was analyzed. (**F**) LPS-tolerant J774A.1 cells were transfected with control siRNA (siNC) or GFAT1 and 2 siRNA (siGFAT) for 48 h and then untreated or treated with LPS alone, or together with Gln for 24 h. Then the cells were cross-linked for 3 h with DSS, and DRP1 oligomer was detected. (**G**) LPS-tolerant J774A.1 cells were treated as in (**F**). Cytosolic calcium ions and mitochondrial calcium ions were detected. (**H**) LPS-tolerant J774A.1 cells were transfected with control siRNA (siNC) or GFAT1 and 2 siRNA (siGFAT) for 48 h and then untreated or treated with Gln for 24 h, then infected with *E. coli* and *S. aureus* for 1 h. Phagocytosis was analyzed. The concentration of LPS, Gln, CB839, and DON was 100 ng/ml, 2 mM, 10 μM and 50 μM, respectively. The MOI for *E. coli* and *S. aureus* was 1. M: Normal macrophages that have not been treated with Gln for (**A**), LPS-tolerant macrophages that have not been treated with Gln for (**F**). **: *P*<0.01, *N.S*.: no significance. *n*=3.

The self-assembly of DRP1 into helical oligomers facilitates its mitochondrial translocation and stimulates its GTPase activity [32]. We next investigated whether Gln affects DRP1 oligomerization, thus promoting its translocation and activation. As expected, Gln significantly increased DRP1 oligomer formation in LPS-tolerant macrophages (Figure 4A). To address the mechanism, we explored pathways of Gln utilization of either conversion into glutamate via glutaminases (GLSs) or participation in the hexosamine biosynthesis pathway (HBP) catalyzed by GFAT [29]. We found that the GLS inhibitor CB839 did not affect DRP1 oligomer formation, whereas the GFAT inhibitor DON significantly suppressed it (Figure 4B and C). Consistently, DON, but not CB839, interfered with the Gln-induced changes in calcium ion dynamics and macrophage phagocytosis (Figure 4D and E and Supplementary Figure S7AB). Consistent with this, another GLS inhibitor BPTES was also unable to affect Gln-induced DRP1 oligomer formation, changes in calcium ion dynamics, and macrophage phagocytosis (Supplementary Figure S7C, D, E). Further confirmation through siRNA-mediated GFAT knockdown showed that reduced GFAT expression diminished Gln-induced DRP1 oligomer formation (Figure 4F). Likewise, cytoplasmic calcium elevation and mitochondrial calcium reduction were significantly abolished, accompanied by the disruption of macrophage phagocytosis (Figure 4G and H and Supplementary Figure S7F,G). Taken together, our data suggest that Gln promotes mitochondrial fission, elevates cytoplasmic calcium levels, and enhances macrophage phagocytosis through GFAT-mediated DRP1 oligomerization.

### Gln supports DRP1 oligomerization by GFAT-mediated O-GlcNAcylation modification

**Figure 5:**
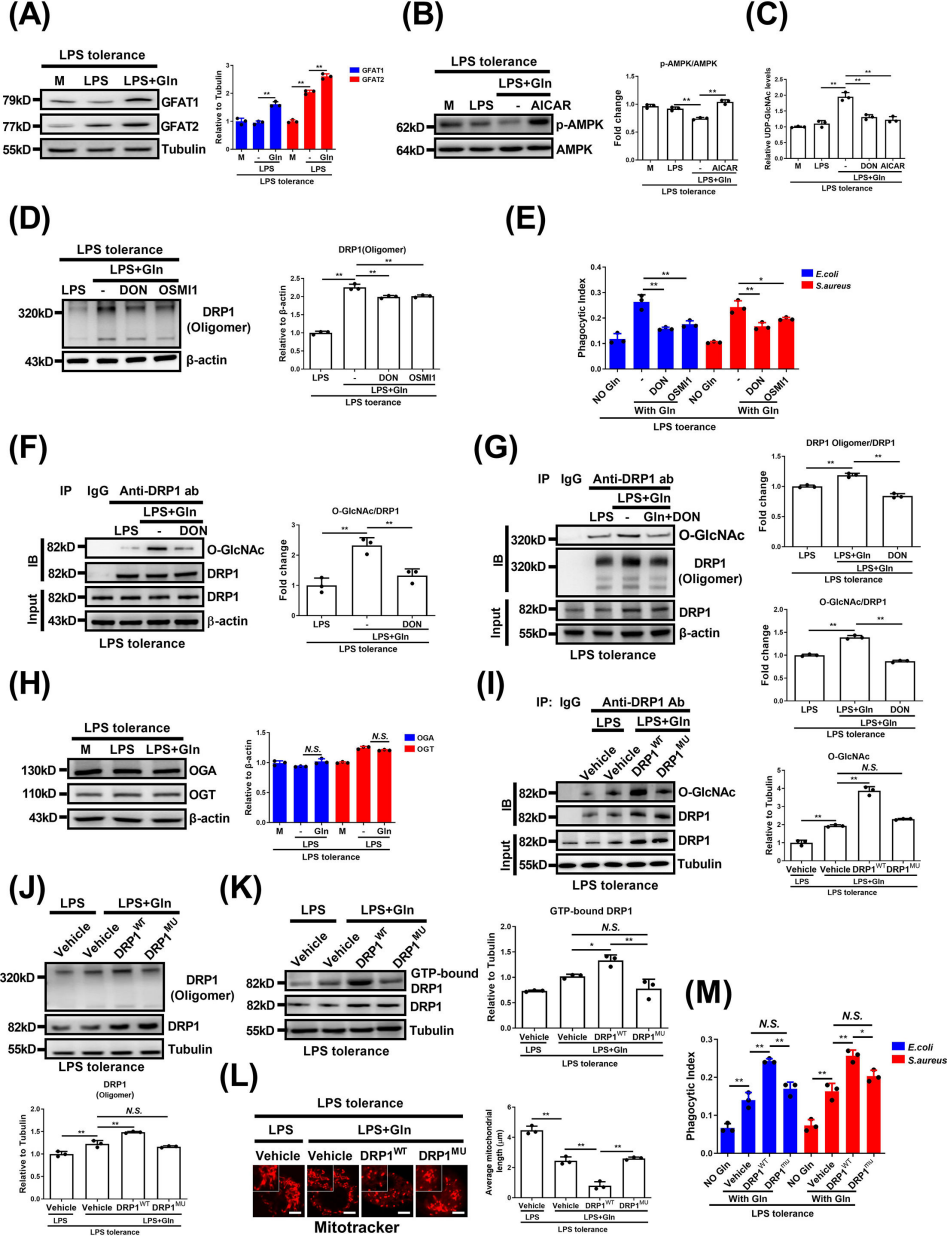
Gln supports DRP1 oligomerization by GFAT-mediated O-GlcNAcylation modification. (**A**) LPS-tolerant BMDMs were untreated or treated with LPS or together with Gln for 24 h. The protein expression of GFAT1 or GFAT2 was detected. (**B-C**) LPS-tolerant BMDMs were treated with LPS or together with Gln, or further treated with AICAR for 24 h. The protein expression of AMPK and p-AMPK (**B**) and the UDP-GlcNAc (**C**) were assayed. (**D-E**) LPS-tolerant BMDMs were treated with LPS alone or together with Gln, or further treated with DON or OSMI1 for 24 h. The DRP1 oligomer (**D**) and the phagocytosis of BMDMs (**E**) were analyzed. (**F**) LPS-tolerant BMDMs were treated with LPS alone or together with Gln, or further treated with DON for 24 h. The amount of O-GlcNAc conjugated with DRP1 was detected. (**G**) LPS-tolerant BMDMs were treated with LPS alone or together with Gln, or further treated with DON for 24 h. The amount of O-GlcNAc conjugated with DRP1 oligomer was detected. (**H**) LPS-tolerant BMDMs were treated with LPS alone or together with Gln for 24 h. The protein expression of OGA and OGT was detected. (**I-M**) LPS-tolerant J774A.1 cells of DRP1^WT^ and DRP1^MU^ overexpression were treated with LPS alone or together with Gln for 24 h. The amount of O-GlcNAc conjugated with DRP1 (**I**), the DRP1 oligomer (**J**), the GTP-binding activity of DRP1 (**K**), the mitochondrial morphology (**L**), and the phagocytosis (**M**) of J774A.1 cells were analyzed. The concentration of LPS, Gln, AICAR, DON, and OSMI1 was 100 ng/ml, 2 mM, 1 mM, 50 μM and 10 μM, respectively. The MOI for *E. coli* and *S. aureus* was 1. M: LPS-tolerant macrophages that have not been treated with Gln. **: *P*<0.01, *: *P*<0.05, *N.S*.: no significance. *n*=3. Scale bar = 5 µm.

Protein O-GlcNAcylation is known to enhance the formation and stability of protein oligomers [33,34]. GFAT is the rate-limiting enzyme of HBP and regulates protein function through O-GlcNAcylation via catalyzing the production of UDP-GlcNAc, an O-GlcNAcylation substrate. Therefore, we next investigated whether Gln facilitates DRP1 oligomerization via GFAT-mediated O-GlcNAcylation. First, we assessed the expression and activity of GFAT. Gln up-regulated GFAT1 and GFAT2 levels in LPS-tolerant macrophages, suggesting a possible transcriptional control (Figure 5A). AMPK negatively regulates GFAT upon activation [35]. Herein, Gln inhibited AMPK phosphorylation, suggesting that it could increase the activity of GFAT (Figure 5B). Mass spectrometry confirmed that Gln elevated UDP-GlcNAc production, which was inhibited by DON or the AMPK agonist AICAR (Figure 5C). Next, we investigated whether Gln promotes DRP1 oligomer formation through GFAT-mediated O-GlcNAcylation. The O-GlcNAc transferase (OGT) inhibitor OSMI1 reduced DRP1 oligomerization and macrophage phagocytosis (Figure 5D and E and Supplementary Figure S8). The immunoprecipitation assay showed that Gln significantly promoted DRP1 O-GlcNAcylation (Figure 5F) and enhanced the O-GlcNAcylation deposited on DRP1 oligomers (Figure 5G). Moreover, DON significantly inhibited the O-GlcNAcylation of both DRP1 and DRP1 oligomers, confirming the dependence of GFAT in mediating DRP1 O-GlcNAcylation (Figure 5F and G). Of note, Gln had no effect on the protein levels of the writer and the eraser of O-Gln did not alter OGT or O-GlcNAcase (OGA) levels, indicating its effects are independent of these enzymes (Figure 5H). To affirm the necessity of O-GlcNAcylation, we transfected DRP1 KD J774A.1 cells with either DRP1^WT^ and O-GlcNAcylation site (Ser-604 and Ser-605 sites) mutant (DRP1^MU^) plasmid. While DRP1 levels rose in both cell types (Supplementary Figure S8B, C7F,G), DRP1^MU^ cells had disrupted DRP1 O-GlcNAcylation (Figure 5I), oligomer formation (Figure 5J) or GTP-binding activity of DRP1 (Figure 5K) in response to Gln treatment, unlike the recovered activity in DRP1^WT^ cells. Mitochondrial fission was not observed in DRP1^MU^ macrophages despite Gln treatment (Figure 5L and Supplementary Figure S8D). Correspondingly, Gln enhanced bacterial phagocytic activity in DRP1^WT^, but not DRP1^MU^ macrophages (Figure 5M and Supplementary Figure S8E). In summary, Gln promotes DRP1 oligomerization and macrophage phagocytosis via GFAT-mediated O-GlcNAcylation.

#### Gln enhances DRP1 Ser-616 phosphorylation through the GFAT-CDK1 axis, independent of GFAT-mediated O-GlcNAcylation or other DRP1-related kinases

**Figure 6:**
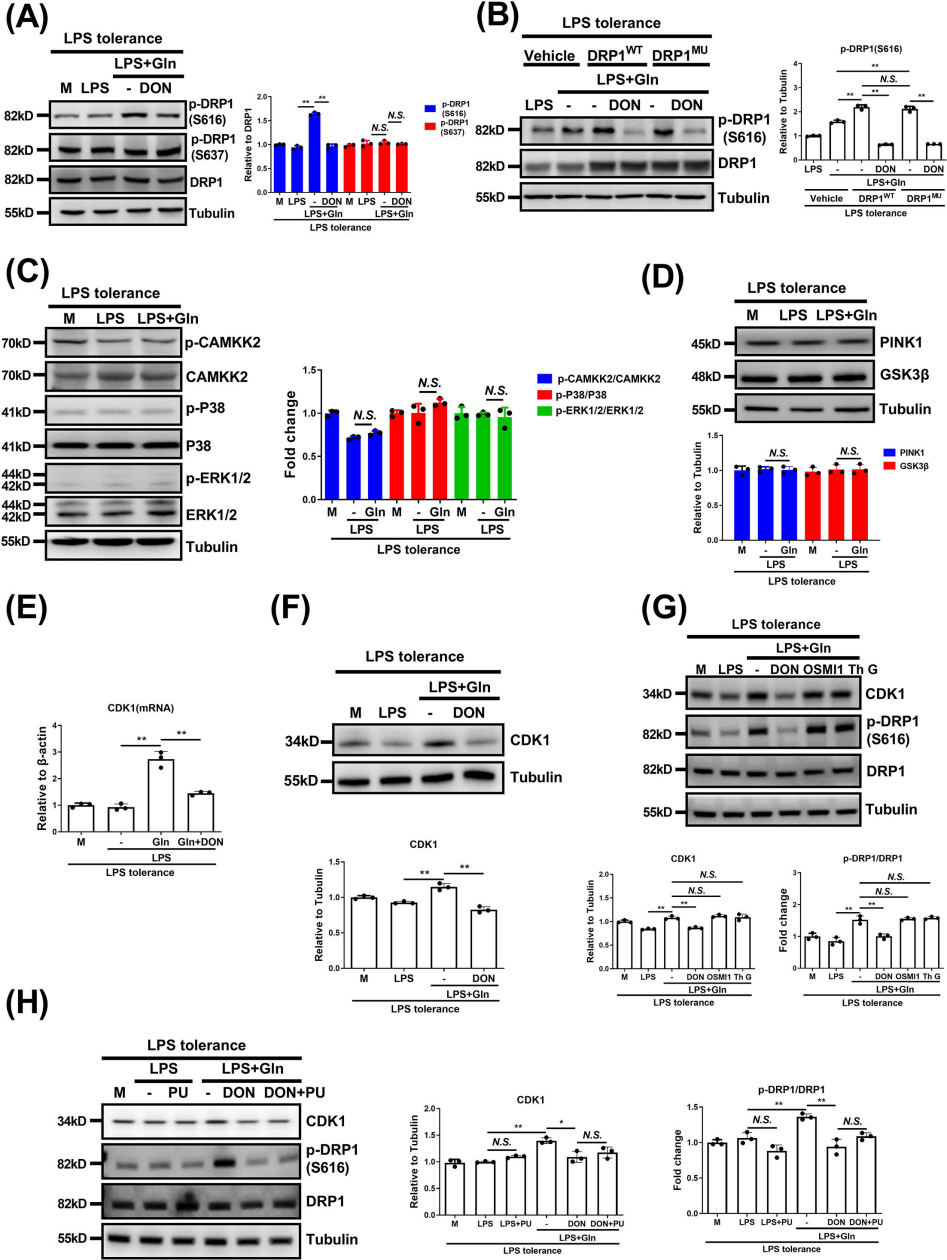
Gln promotes DPR1 phosphorylation by elevating CDK1 protein expression, independent of GFAT-mediated O-GlcNAcylation. (**A**) LPS-tolerant BMDMs were untreated or treated with LPS or together with Gln, or further treated with DON for 24 h. The protein expression of DRP1 or phosphorylated DRP1 at site Ser-616 or Ser-637 [p-DRP1 (S616) and p-DRP1 (S637)] was detected. (**B**) LPS-tolerant DRP1^WT^ and DRP1^MU^ J774A.1 cells were treated with LPS or together with Gln, or further treated with DON for 24 h. The protein expression of DRP1 and p-DRP1(S616) was detected. (**C-D**) LPS-tolerant BMDMs were untreated or treated with LPS or together with Gln for 24 h. The protein expression of CAMKK2, phosphorylated CAMKK2 (p-CAMKK2), P38, phosphorylated P38 (**p-P38**), ERK1/2, phosphorylated ERK1/2 (p-ERK1/2) (**C**) and PINK1, GSK3β (**D**) was detected. (**E-F**) LPS-tolerant BMDMs were treated as in (**A**). The mRNA (**E**) and protein (**F**) expression of CDK1 was detected. (**G**) LPS-tolerant BMDMs were untreated or treated with LPS alone or together with Gln, or further treated with DON, OSMI1, or Thiamet G (Th G) for 24 h. The protein expression of CDK1, DRP1, and p-DRP1 (S616) was detected. (**H**) LPS-tolerant BMDMs were untreated or treated with LPS alone or together with Gln, or further treated with PUGNAc (PU) for 24 h. The protein expression of CDK1, DRP1, and p-DRP1 (S616) was detected. The concentration of LPS, Gln, DON, OSMI1, Thiamet G, and PUGNAc was 100 ng/ml, 2 mM, 50 μM, 10 μM, 10 μM, and 100 μM, respectively. The MOI for *E. coli* and *S. aureus* was 1. M: LPS-tolerant macrophages that have not been treated with Gln. **: *P*<0.01, *: *P*<0.05, *N.S*.: no significance. *n*=3.

Phosphorylation at Ser-616 activates DRP1, while Ser-637 inhibits it [16]. Therefore, we next examined Gln’s effect on DRP1 phosphorylation. Western blot results showed Gln increased phosphorylation at Ser-616, with no effect on Ser-637, and this was inhibited by DON (Figure 6A). Despite typical cross-talk between O-GlcNAcylation and phosphorylation at serine/threonine sites [34], Gln enhanced DRP1 Ser-616 phosphorylation in both wildtype (DRP1^WT^) and mutant (DRP1^MU^) macrophages, unaffected by O-GlcNAcylation status but inhibited by DON (Figure 6B). This suggests GFAT modulates this phosphorylation independent of O-GlcNAcylation. Moreover, Gln did not affect the phosphorylation of CAMKK2, P38, and ERK1/2 (Figure 6C) or the protein expression of PINK1 and GSK3β (Figure 6D), excluding the involvement of these upstream molecules that catalyze the phosphorylation of DRP1 [36–40]. CDK1 was recently shown to induce DRP1 Ser-616 phosphorylation [41]. Notably, Gln significantly increased CDK1 mRNA and protein levels, inhibited by DON, indicating GFAT dependence (Figure 6E and F). Neither OGT nor OGA inhibitors affected Gln’s ability to promote CDK1 expression and DRP1 phosphorylation (Figure 6G). Treatment with an O-GlcNAcylation agonist PUGNAc also failed to promote the expression of CDK1 protein and phosphorylation of DRP1 in LPS-tolerant macrophages. Meanwhile, regardless of the presence or absence of PUGNAc, DON significantly inhibited the effect of Gln to promote CDK1 protein expression and DRP1 Ser-616 phosphorylation (Figure 6H).

**Figure 7:**
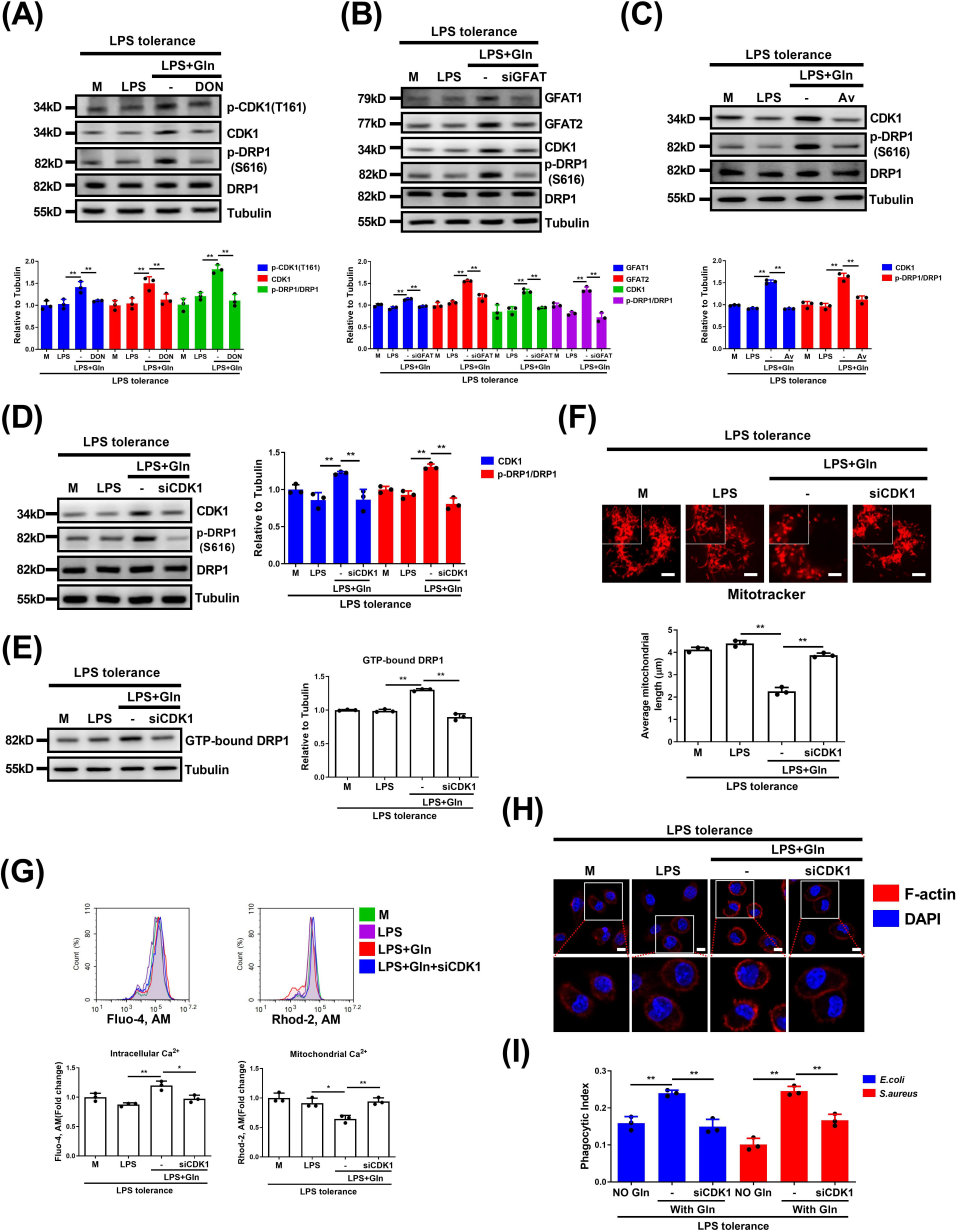
Gln elevates DRP1 Ser-616 phosphorylation, mitochondrial fission, and phagocytosis via GFAT-CDK1 axis. (**A**) LPS-tolerant BMDMs were untreated or treated with LPS or together with Gln or further treated with DON for 24 h. The protein expression of CDK1, phosphorylated CDK1 at site Thr-161 [p-CDK1 (T161)] DRP1 or p-DRP1(S616) was detected. (**B**) LPS-tolerant J774A.1 cells were transfected with control siRNA (siNC) or GFAT1 and two siRNA (siGFAT) for 48 h and then untreated or treated with LPS alone, or together with Gln for 24 h. The protein expression of GFAT1, GFAT2, CDK1, DRP1, or p-DRP1 (S616) was detected. (**C**) LPS-tolerant BMDMs were untreated or treated with LPS or together with Gln or further treated with Avotaciclib (Av) for 24 h. The protein expression of CDK1, DRP1, or p-DRP1 (S616) was detected. (**D-H**) LPS-tolerant J774A.1 cells were transfected with control siRNA (siNC) or CDK1 siRNA (siCDK1) for 48 h and then untreated or treated with LPS alone, or together with Gln for 24 h. The protein expressions of CDK1, DRP1, or p-DRP1(S616) (**D**), GTP-binding activity of DRP1 (**E**), mitochondrial morphology (**F**), cytosolic calcium ions, mitochondrial calcium ions (**G**), and fluorescence images of intracellular F-actin were detected (**H**). (**I**) LPS-tolerant J774A.1 cells were transfected with control siRNA (siNC) or CDK1 siRNA (siCDK1) for 48 h and then untreated or treated with LPS alone, or together with Gln for 24 h, and then infected with *E. coli* and *S. aureus* for 1 h. Phagocytosis was analyzed. The concentration of LPS, Gln, DON, and Avotaciclib was 100 ng/ml, 2 mM, 50 μM and 5 μM, respectively. The MOI for *E. coli* and *S. aureus* was 1. M: LPS-tolerant macrophages that have not been treated with Gln. **: *P*<0.01, *: *P*<0.05, *N.S*.: no significance. *n*=3. Scale bar = 5 µm.

Phosphorylation at Thr-161 of CDK1 is essential to support its activity [42]. Our subsequent experiments showed that Gln up-regulated the expression of CDK1 while significantly enhancing the phosphorylation of CDK1 Thr-161 (Figure 7A), suggesting that Gln promotes CDK1 activation by increasing its expression. Notably, GFAT inhibition via DON or GFAT knockdown reduced Gln’s effect on CDK1 and DRP1 Ser-616 phosphorylation (Figure 7A and B). To further clarify that DRP1 Ser-616 phosphorylation is mediated by CDK1, Avotaciclib hydrochloride or siRNA was used to respectively inhibit CDK1 or down-regulate its expression. Both Avotaciclib hydrochloride and CDK1-specific siRNA treatment significantly reduced the effect of Gln on promoting DRP1 Ser-616 phosphorylation (Figure 7C and D). Furthermore, the knockdown of CDK1 also significantly reduced the GTP-binding activity and mitochondrial fission induced by Gln (Figure 7E and F and Supplementary Figure S9A). CDK1 deficiency inhibited Gln-induced cytosolic calcium increase, restoring mitochondrial calcium overload, actin polymerization, and suppressed macrophage phagocytosis enhancement (Figure 7G, H1 and Supplementary Figure S9B). Consistently, overexpression of CDK1 enhanced the DRP1 GTPase activity and cytoplasmic calcium, suppressed mitochondrial calcium, and enhanced macrophage phagocytosis induced by Gln. However, this effect of CDK1 overexpression was suppressed by mitochondrial fission inhibitor Mdivi-1, which further suggests DRP1 as a key downstream substrate for CDK1 (Supplementary Figure S9C,D,E). A study has shown that CDK1 can phosphorylate inositol-1,4,5-triphosphate receptors (IP3Rs), thereby promoting calcium ion release into the cytoplasm [43]. However, we found that the endoplasmic reticulum calcium ion channel IP3Rs inhibitor 2-APB had no effect on the Gln-induced increase in cytoplasmic calcium or the decrease in mitochondrial calcium. Conversely, the mitochondrial Na^+^/Ca^2+^ exchanger (NCLX) inhibitor CGP37157 (CGP) significantly inhibited both the Gln-induced cytoplasmic calcium increase and the decrease in mitochondrial calcium (Supplementary Figure S9F). Taken together, our data show that the GFAT-CDK1 axis drives Gln-induced mitochondrial fission and macrophage phagocytosis by phosphorylating DRP1 at Ser-616.

### Gln alleviates septic immunosuppression via the GFAT-CDK1-DRP1 axis

**Figure 8:**
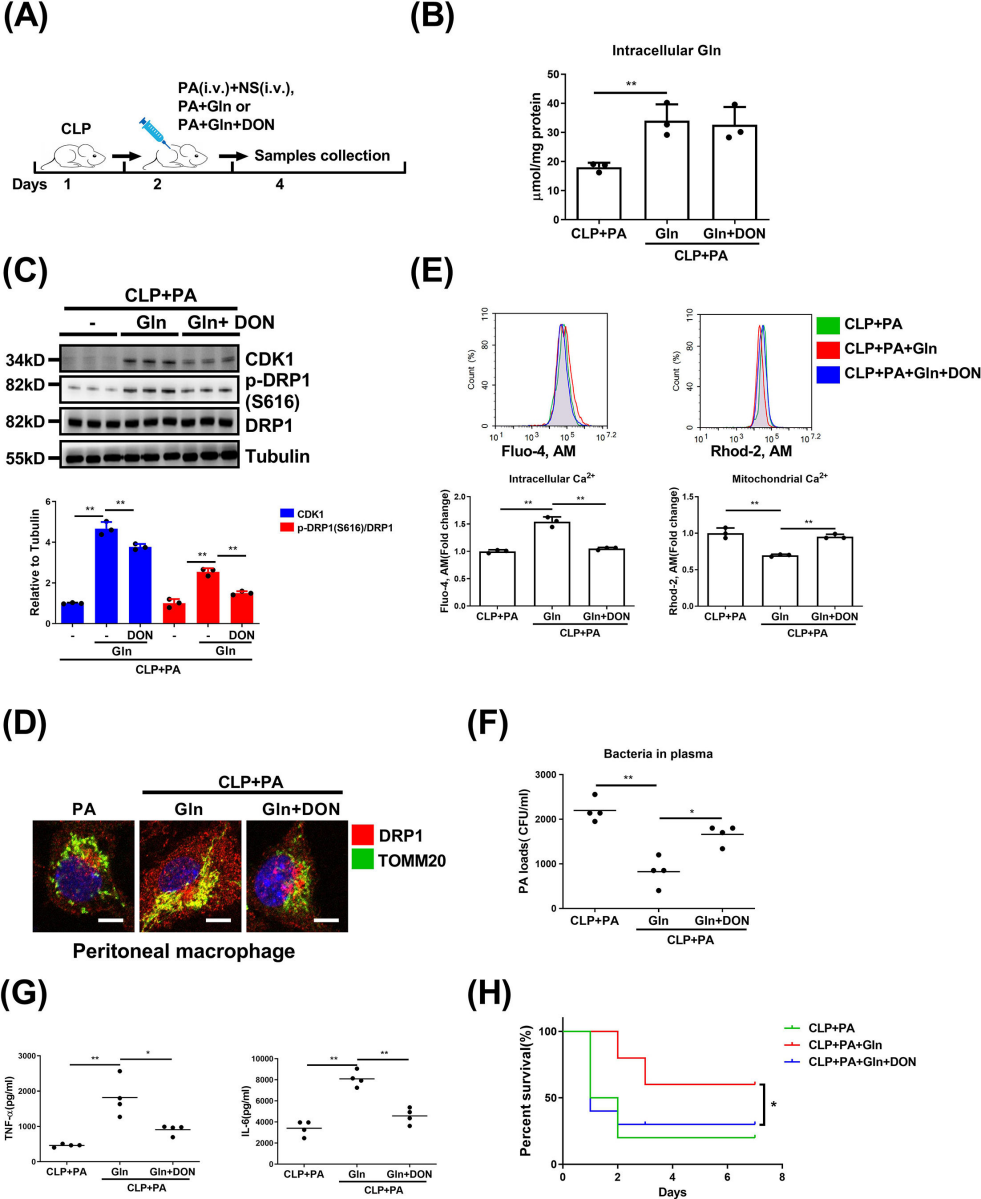
Gln alleviates septic immunosuppression via the GFAT-CDK1-DRP1 axis (**A-H**) BALB/c mice were infected with *P. aeruginosa* (PA, 1 × 109 CFU/kg body weight) 24 h after CLP surgery, and the mice were treated with normal saline (NS) or Gln (0.1 g/kg body weight) or together with DON (3 µg/kg body weight) every 24 h (**A**). After 48 h, the intracellular Gln concentration of peritoneal macrophages (**B**), protein expression of CDK1, DRP1 and p-DRP1 (S616) in peritoneal macrophages (**C**), intracellular co-localization of DRP1 and TOMM20 in peritoneal macrophages (**D**), cytosolic calcium ions and mitochondrial calcium ions (**E**), PA load (**F**) and TNF-α and IL-6 in serum (**G**) were analyzed. The survival rate of mice was observed (**H**) (*n*=10). **: *P*<0.01, *: *P*<0.05, *N.S*.: no significance. *n*=3. Scale bar = 5 µm.

Our initial experiments in the present study demonstrated that Gln could alleviate sepsis immunosuppression by augmenting macrophage phagocytosis (Figure 1), and the specific mechanism has also been elaborated *in vitro* (Figures 2–7). To further confirm the role of Gln in alleviating immune suppression in sepsis by regulating macrophage phagocytosis through the GFAT-CDK1-DRP1 axis, we conducted additional *in vivo* experiments by giving CLP mice Gln alone or together with DON treatment after a secondary bacterial injection (Figure 8A). The intracellular Gln concentration of peritoneal macrophages was significantly increased in the group treated with Gln alone (Figure 8B), while this significantly promoted the expression of CDK1 and the phosphorylation (Ser-616) of DRP1 in isolated peritoneal macrophages (Figure 8C). Although the intracellular Gln in peritoneal macrophages was not altered by further DON treatment (Figure 8B), DON significantly inhibited the protein expression of CDK1 and the phosphorylation (Ser-616) of DRP1 (Figure 8C). By using the immunofluorescent assay, we observed that Gln treatment significantly enhanced the co-localization of DRP1 and TOMM20, suggesting that Gln promotes the translocation of DRP1 to mitochondria, while DON significantly inhibited this process (Figure 8D). Furthermore, Gln significantly increased intracellular calcium levels while decreasing mitochondrial calcium in peritoneal macrophages of model mice, whereas this effect was markedly inhibited by co-treatment with DON (Figure 8E). In accordance with these results, Gln also significantly reduced the bacterial load in model mice, while DON co-treatment resulted in increased bacterial counts (Figure 8F). Consistently, the inflammatory mediators in the serum were also significantly reduced (Figure 8G), and the survival rate was also markedly decreased (Figure 8H) by DON co-treatment, compared with the group Gln given alone. In summary, Gln increases the expression of CDK1 in a GFAT-dependent manner, thereby increasing DRP1 phosphorylation (Ser-616) and its activity. This promotes mitochondrial fission and the subsequent calcium-mediated macrophage phagocytosis, thereby effectively clearing pathogens and improving the survival rate of immunosuppressive mice under secondary bacterial attacks.

## Discussion

**Figure 9:**
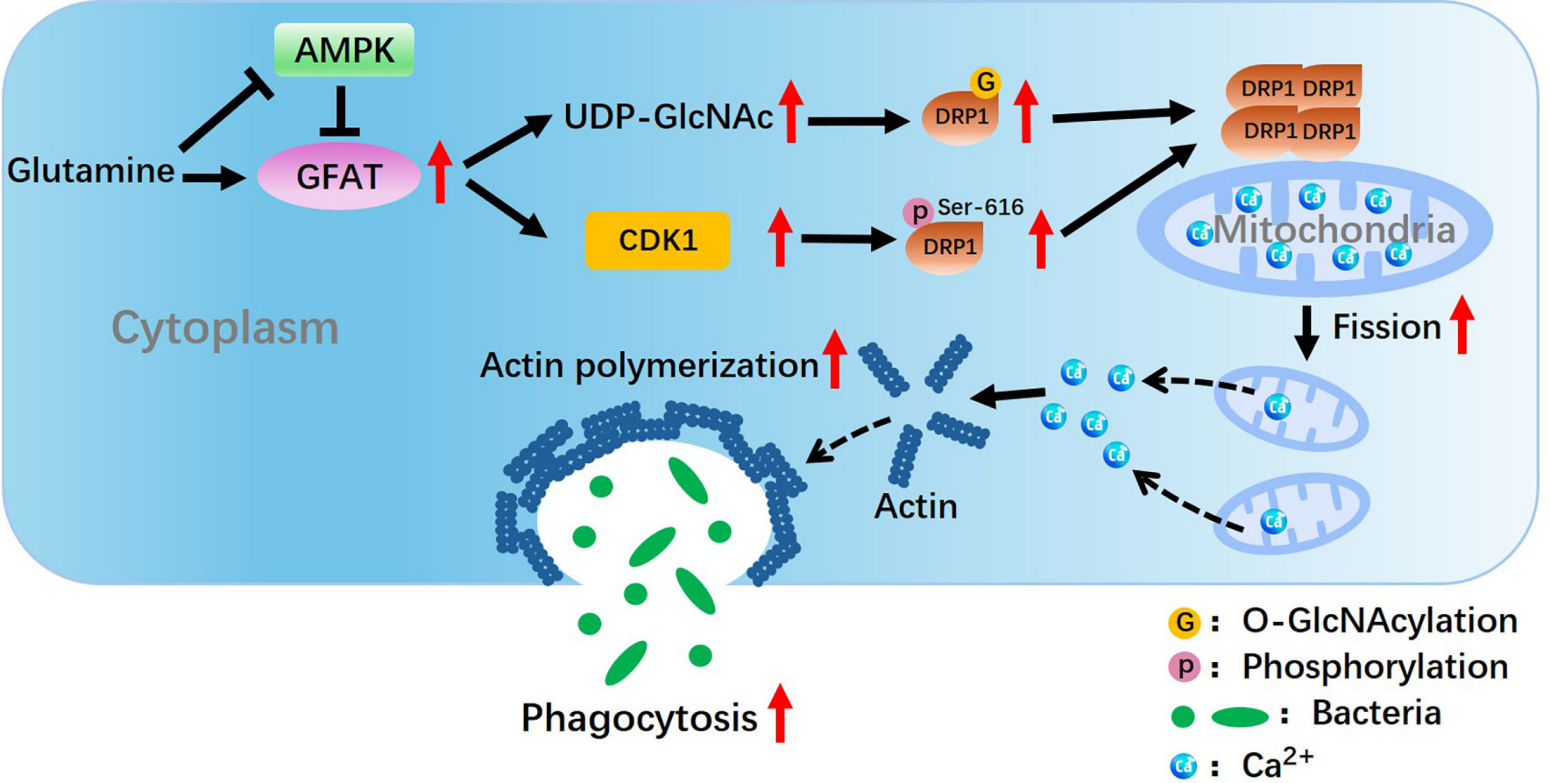
Schematic model depicting a mechanism of Gln promoting mitochondrial fission and rescuing the impaired macrophage phagocytosis in sepsis-induced immunosuppression.

Integrating metabolic control with signaling pathways is crucial for restoring impaired phagocytosis in sepsis, though the linking mechanisms remain unclear. Our study reveals that Gln can reverse compromised macrophage phagocytosis and mitigate immunosuppression in polymicrobial sepsis. This effect is due to the targeted induction of DRP1-dependent mitochondrial fission and calcium release, which maintains cytosolic calcium levels necessary for phagocytosis (Figure 9). We show that Gln promotes GFAT-dependent protein O-GlcNAcylation, enhancing DRP1 oligomerization critical for its translocation and activation. Additionally, we uncover a novel GFAT-CDK1-DRP1 axis that increases DRP1 Ser-616 phosphorylation, further activating it. Taken together, the present study suggests potential pharmaceutical values for promoting macrophage phagocytosis and treating sepsis-associated immunoparalysis by targeting Gln availability and Gln-associated GFAT pathways.

In our initial experiments, we observed a robust decline of Gln in the immunosuppressive phase of a classical murine model of sepsis, which is in agreement with the profound Gln deficit in critically ill patients [29]. The rapid decrease in Gln is reasonable, as immune cells prefer to utilize Gln at high rates in catabolic conditions such as sepsis [29]. Meanwhile, a compromised availability of Gln due to impairment in its synthesis may worsen the already appeared Gln shortage [29]. Of note, it was previously observed that a reduction in Gln supplementation in culture medium from 0.6 to 0.05 mM greatly impaired proliferation of human lymphocytes and phagocytosis in mouse macrophages [23]. Other studies, along with those performed in our lab, have also evaluated the contribution of Gln supply in sustaining the survival, proliferation, activation, and function of immune cells in critical illness [30]. Here we report an additional therapeutic effect of Gln supply in a condition of sepsis-associated immunosuppression. These findings may together support the importance of extra Gln supply to reverse its insufficiency and rescue the compromised immune response. In view of bacterial killing as the primary biological feature in this stage, our research focused on its ability to reduce bacterial load and increase survival. We also confirmed this effect to be associated with macrophage bacterial phagocytosis. Therefore, our consideration to increase the availability of Gln in immunosuppressive conditions may strengthen the beneficial concept of immune-based effects for glutamine supplementation in treating sepsis.

An abundant expression of phagocytic receptors couples with intracellular signaling pathways to orchestrate the phagocytic machinery, allowing macrophages to efficiently capture and engulf pathogens. Our study found that Gln doesn’t affect the expression of these receptors but instead facilitates actin remodeling by maintaining cytosolic calcium levels. This finely tuned regulation is crucial, as cytosolic calcium links receptor sensing to downstream signaling that initiates actin remodeling and phagosome maturation [11,15,44]. Our findings suggest a novel interaction between nutrients and secondary signals that supports bacterial phagocytosis. Mitochondria play a pivotal role in buffering intracellular calcium by regulating calcium uptake and efflux. Mitochondrial calcium efflux disorders, seen in murine cardiac tissues during sepsis, lead to calcium overload and worsen organ injury [45,46]. Here we strengthened this concept by demonstrating that impaired mitochondrial calcium efflux can also hinder macrophage phagocytosis and trigger immune dysfunction in sepsis. Interestingly, we discovered that Gln elevates intracellular calcium specifically by promoting mitochondrial efflux. This action prevents mitochondrial calcium overload, reducing the risk of cell death, while restoring intracellular calcium to enhance phagocytosis and cellular immunity. Our results align with a recent study showing that increased cytosolic calcium is crucial for macrophage phagocytosis, which can be compromised when Gln is redirected to tumors [15]. Overall, these findings highlight Gln’s conserved role in stimulating immunity by enhancing mitochondrial calcium release, thereby supporting macrophage phagocytic activity against tumor cells or invading pathogens.

It is known that extrusion of calcium from mitochondria is intimately connected with the dynamin-like GTPase DRP1-executed mitochondrial fission, a process that can either augment phagocytosis of liver tumor cells [15] or otherwise promote efferocytosis of multiple apoptotic cells [16]. Inspired by these findings, we speculated that Gln may promote mitochondrial calcium release and sustain calcium-dependent phagocytosis by regulating the DRP1-dependent mitochondrial fission. As expected, our results demonstrated that Gln-starved tolerant macrophages displayed impaired translocation and activity of DRP1, which can be reversed by restoring the Gln supply. We also positively linked Gln availability with the sustaining of mitochondrial fission and calcium efflux that drive macrophage phagocytosis. Of note, recent work has provided opposite results by showing that 1) Gln promotes DRP1-dependent mitochondrial fission in macrophages [15]; 2) Gln-starved tumor cells displayed elevated DRP1 Ser-616 phosphorylation and augmented mitochondrial fragmentation [47]. These apparent discrepancies may be attributed to the cell type difference. In addition, the specific cell condition and demand of Gln may also explain the contradictory findings. However, it is important for further investigation to fully understand the possible ‘double-edge’ role of Gln in orchestrating DRP1 activation and mitochondrial fission.

Gln is primarily converted into glutamate and then α-ketoglutarate by an oxidation process called glutaminolysis [29]. Gln can also be utilized in the hexosamine biosynthesis by its rate-limiting enzyme GFAT. By using chemical inhibitors and genetic ablation approaches to interfere with the Gln metabolic pathways, we excluded possible involvement of the canonical Gln decomposition pathway while identifying the alternative HBP pathway that might mediate DRP1-dependent mitochondrial fission in macrophages. We also confirmed that Gln could up-regulate expression of the rate-limiting enzyme GFAT and thereby augment the HBP pathway to support mitochondrial calcium release and bacterial phagocytosis. These results are supported by a recent study that similarly suggests a GFAT-dependent enhancement of mitochondrial fission and macrophage phagocytosis [15]. However, our data additionally show the role of Gln to both activate the checkpoint enzyme and serve as the key substrate in the HBP, thereby deciphering the regulatory pattern in a more comprehensive manner.

Although Gln was shown recently to sustain mitochondrial fission in a GFAT-dependent manner, the connection between GFAT and DRP1 was not clearly elucidated in this study [15]. To address this concern, we performed subsequent experiments to identify the precise mechanisms that link Gln and GFAT with DRP1 and DRP1-dependent mitochondrial fission. The oligomerization of DRP1 is indispensable for its complete activation [48,49]. Meanwhile, compelling evidence has demonstrated the role of O-GlcNAcylation to augment the formation and maintain the stability of oligomerized proteins, such as the heterodimer of NF-κB in microglia cells and the tetramerization of sterile alpha motif domain and histidine-aspartic domain-containing protein 1 in hepatocytes [50]. In addition, GFAT is known as the rate-limiting enzyme of HBP, which achieves regulation of protein function mainly through O-GlcNAcylation modification of proteins by providing the final product of UDP-GlcNAc as an O-GlcNAcylation substrate [29]. Therefore, it is reasonable to investigate whether Gln promotes DRP1 oligomer formation through GFAT-mediated O-GlcNAcylation modification. As expected, Gln facilitates DRP1 oligomerization through GFAT and O-GlcNAcylation of DRP1. By using site-directed mutation, we also identified two key serine residues (Ser-604 and Ser-605 sites) as the major sites for O-GlcNAcylation modification on DRP1 that mediate the series of actions by Gln. In summary, our results confirm the concept of interplay between O-GlcNAcylation and protein oligomerization while highlighting O-GlcNAcylation as a previously undefined Post-translational modification (PTM) mechanism driven by Gln to promote DRP1 oligomerization and sustain its immunomodulatory activity.

O-GlcNAcylation commonly occupies serine/threonine residues, where its cross-talk with other PTMs, such as phosphorylation, may extensively occur [34]. Indeed, phosphorylation of DRP1 also plays an important role in regulating its activity, with two major phosphorylation sites that either promote (Ser-616) or suppress (Ser-637) its activation [50]. These data thus suggest a possible interplay between protein O-GlcNAcylation and phosphorylation that co-ordinately regulates the DRP1 activation. In the present study, we confirmed that Gln selectively promoted phosphorylation of DRP1 at Ser-616, whereas it had no effect on the inhibitory Ser-637 phosphorylation in macrophages. This effect was mediated by GFAT but independently with its catalyzed protein O-GlcNAcylation. The unexpected findings thus inspired us to further explore the underlying mechanisms and screened out CDK1 as the candidate upstream kinase. As previously identified, CDK1 directly catalyzes DRP1 Ser-616 phosphorylation and thus promotes mitochondrial hyperfragmentation [41]. In another study, Gln was shown to promote CDK1 protein expression in intestinal epithelial cells through mTORC1 activation mediated by phospholipase D [51]. We similarly demonstrated that Gln up-regulates the expression of CDK1 protein, which subsequently induces the phosphorylation of DRP1 in LPS-tolerant macrophages in a GFAT-dependent manner. To further confirm that the CDK1–DRP1 axis is the mechanistic basis of Gln-induced mitochondrial calcium balance and phagocytosis restoration, we performed a series of complementary genetic and pharmacological interventions. These experiments collectively demonstrate that: 1) DRP1 acts as an essential downstream substrate of CDK1 that drives mitochondrial fission and phagocytosis induced by Gln; 2) another CDK1 substrate, IP3R, implicated in organellar calcium flux at the endoplasmic reticulum, was not required for regulating calcium release and phagocytic function. Although we cannot fully exclude the contribution of other CDK1-dependent pathways, these data indicate that the CDK1–DRP1 axis plays a central and non-redundant role in Gln-mediated immunometabolic reprogramming during sepsis. Taken together, our results thus suggest an additional non-enzymatic or non-canonical activity of GFAT rather than merely catalyzing the endpoint product of UDP-GlcNAc, which serves as the substrate for protein O-GlcNAcylation. However, the detailed mechanism by which Gln and GFAT up-regulate CDK1 expression is an important topic deserving further investigation.

To further explore the therapeutic efficacy by targeting the Gln-mediated GFAT pathway, we administered a chemical inhibitor of GFAT in Gln-treated CLP mice. Inhibition of GFAT substantially blocks the efficacy of Gln to enhance phagocytosis and rescue immunoparalysis in septic mice. Moreover, we also confirmed that glutamine can up-regulate CDK1 expression and enhance DRP1 phosphorylation (Ser-616) and its activity, thereby triggering mitochondrial fission and subsequent calcium ion-mediated macrophage phagocytosis. These *in vivo* results not only support data obtained from the *in vitro* experiments but also suggest the GFAT-CDK1-DRP1 axis as a putative therapeutic target in treating sepsis-associated immunosuppression.

The present study has several limitations. First, our conclusions are derived from pre-clinical models and lack validation in human patient samples, which limits its direct clinical translation. Second, the statistical power was constrained by a moderate sample size, while the use of parameter analysis on small sample sets may increase the risk of statistical errors. Future studies with larger cohorts and more robust methods are needed. Third, while we propose a GFAT-CDK1-DRP1 axis in cellular experiments, our *in vivo* verification was mainly obtained from pharmacological study by using a GFAT chemical inhibitor without more direct mechanistic validation via genetic knockout or rescue experiments. Finally, the precise mechanism by which GFAT regulates CDK1 protein expression remains unclear and necessitates further investigation.

**Clinical perspectives**

- Sepsis is a life-threatening clinical syndrome induced by infection and remains the leading cause of ICU mortality worldwide. Sepsis triggers impaired macrophage bacterial phagocytosis, resulting in the inability of the host to control secondary infection, a manifestation known as sepsis-associated immunosuppression. Gln is a widely recognized nutrient with immunomodulatory activity in critical illness. However, a mechanistic link between Gln availability and innate immune responses, such as phagocytosis, has not yet been fully established.
- The present demonstrates that Gln can reverse sepsis-induced immunosuppression by restoring macrophage phagocytic function. This effect is achieved through the selective induction of dynamin-related protein 1 (DRP1)-dependent mitochondrial fission and mitochondrial calcium release, which sustains cytosolic calcium dynamics essential for bacterial phagocytosis. Mechanistically, Gln promotes both O-GlcNAcylation-dependent and -independent pathways via Gln-fructose-6-phosphate transaminase, which thereby facilitates the oligomerization and phosphorylation of DRP1, ultimately leading to its activation.
- Our study highlights the clinical potential of Gln availability and its metabolic pathway as druggable targets to treat immune deactivation in sepsis.

## Supplementary material

Online supplementary material 1

## Data Availability

The authors declare that all the data supporting the findings of this study are available within the article and its supplementary.

## Open Access

This article has been published open access under our Subscribe to Open programme, made possible through the support of our subscribing institutions, learn more here: https://portlandpress.com/pages/open_access_options_and_prices#conditional

## Abbreviations

E. coli: Escherichia coli
S. aureus: Staphylococcus aureus
P. aeruginosa: Pseudomonas aeruginosa
IP3Rs: inositol-1,4,5-triphosphate receptors
ACN: acetonitrile
AICAR: 5-Aminoimidazole-4-carboxamide-1-β-D-ribofuranoside
ANOVA: analysis of Variance
BALB/c: bagg’ s albino/c
BPTES: (1Z, 1’Z)-N’, N’’-(5, 5’-(thiobis(ethane-2, 1-diyl))bis(1, 3, 4-thiadiazole-5, 2-diyl))bis(2-phenylacetimidic acid)
BSA: bovine serum albumin
CDK1: cyclin-dependent kinase 1
CFU: colony-forming units
CLP: cecum ligation and puncture
COPD: chronic obstructive pulmonary disease
DMEM: Dulbecco’s Modified Eagle’s Medium
DON: 6-Diazo-5-oxo-L-nor-leucine
DRP1: dynamin-related protein 1
DRP1^MU^: glycosylation site mutations DRP1
DRP1^WT^: DRP1 wildtype
DSS: disuccinimidyl suberate
EGTA: ethylene glycol-bis(2-aminoethylether)-N, N, N′, N′-tetraacetic acid
ELISA: enzyme-linked immunosorbent assay
EP: eppendorf
*E. coli*: *Escherichia coli*
FBS: fetal bovine serum
GFAT: glutamine-fructose-6-phosphate transaminase
GLSs: glutaminases
Gln: glutamine
HBP: hexosamine biosynthesis pathway
HILIC: hydrophilic Interaction Liquid Chromatography
IP3Rs: inositol-1,4,5-triphosphate receptors
LPS: lipopolysaccharides
LSCM: laser scanning confocal microscopy
MFI: mean fluorescence intensity
MOI: multiplicity of infection
NCLX: mitochondrial Na^+^/Ca^2+^ exchanger
OGA: O-GlcNAcase
OGT: O-GlcNAc transferase
O-GlcNAc: O-linked N-acetylglucosamine
OSMI-1: (R)-N-(furan-2-ylmethyl)-2-(2-methoxyphenyl)-2-(2-oxo-1, 2-dihydroquinoline-6-sulfonamido)-N-(thiophen-2-ylmethyl)acetamide
PTM: post-translational modification
PUGNAc: O-(2-acetamido-2-deoxy-D-glucopyranosylidene) amino N-phenyl carbamate
PVDF: polyvinylidene fluoride
*P. aeruginosa*: *Pseudomonas aeruginosa*
SIC: selected ion chromatogram
SR-AI: scavenger receptor class A type I
SR-BI: scavenger receptor class B type I
TBST: Tris-buffered saline with Tween 20
TEM: transmission electron microscopy
TLR: toll-like receptor
UHPLC: ultra High Performance Liquid Chromatography;
