# Supplementary material for: Glutamine alleviates immunosuppression in polymicrobial sepsis by augmenting bacterial phagocytosis through sustaining the GFAT-DRP1 dependent mitochondrial calcium dynamics
Source: Clin Sci (Lond). 2025 Oct 22;139(20):CS20256651. doi: 10.1042/CS20256651 (PMC12687432; doi:10.1042/CS20256651)
Supplement: Online supplementary material 1 [file cs-139-20-CS20256651-s001.pdf]

Supplementary materials

Table S1. Sequence of PCR primers

Name	Primer Sequence
CD16	F: 5’-AATGCACACTCTGGAAGCCAA-3’
	R: 5’-CACTCTGCCTGTCTGCAAAAG-3’
CD32	F: 5’-GGAATCCTGCCGTTCCTACTG-3’
	R: 5’-ATGGCACAAAGTCCGTGAGAA-3’
CD64	F: 5’-GTCGGTGGGGAAGTGGTTAAT-3’
	R: 5’-CCCCTCACACCATAAAGTGAC-3’
CD14	F: 5’-ACTTCTCAGATCCGAAGCCAG-3’
	R: 5’-CCGCCGTACAATTCCACAT-3’
TLR2	F: 5’-CTCTTCAGCAAACGCTGTTCT-3’
	R: 5’-GGCGTCTCCCTCTATTGTATTG-3’
TLR4	F: 5’-ATGGCATGGCTTACACCACC-3’
	R: 5’-GAGGCCAATTTTGTCTCCACA-3’
SR-AI	F: 5’-TGGAGGAGAGAATCGAAAGCA-3’
	R: 5’-CTGGACTGACGAAATCAAGGAA-3’
SR-BI	F: 5’-TTTGGAGTGGTAGTAAAAAGGGC-3’
	R: 5’-TGACATCAGGGACTCAGAGTAG-3’
CDK1	F: 5’-AGGTACTTACGGTGTGGTGTAT-3’
	R: 5’-CTCGCTTTCAAGTCTGATCTTCT-3’
TNF-α	F: 5’-CAGGCGGTGCCTATGTCTC-3’
	R: 5’-CGATCACCCCGAAGTTCAGTAG-3’
IL-6	F: 5’-CTGCAAGAGACTTCCATCCAG-3’
	R: 5’-AGTGGTATAGACAGGTCTGTTGG-3’
β-actin	F: 5’-GGGAAATCGTGCGTGACATCAAAG-3’
	R:5’-CATACCCAAGAAGGAAGGCTGGAA-3’

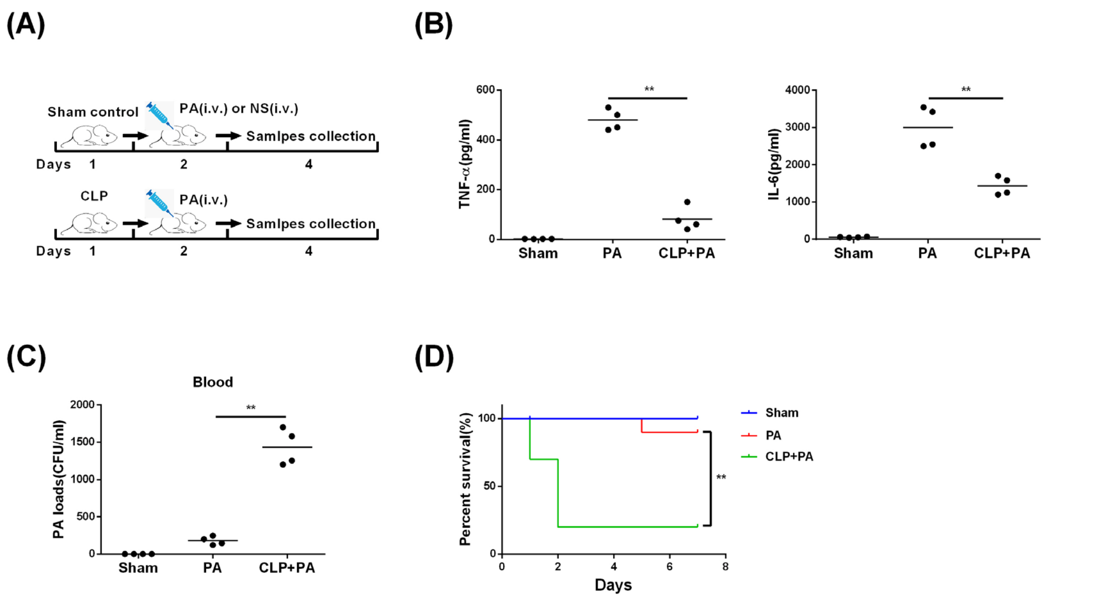

Figure S1. Establishment of sepsis induced immunosuppression model

(**A-D**) BALB/c mice were infected with *P. aeruginosa* (PA, 1×10^9 CFU/kg body weight) 24 h after CLP surgery (**A**). After 48 h, TNF-α and IL-6 in serum (**B**), PA load (**C**) and the 7-day survival rate of mice (**D**) was detected (n = 10). **: P<0.01. n = 4 for (**A-C**).

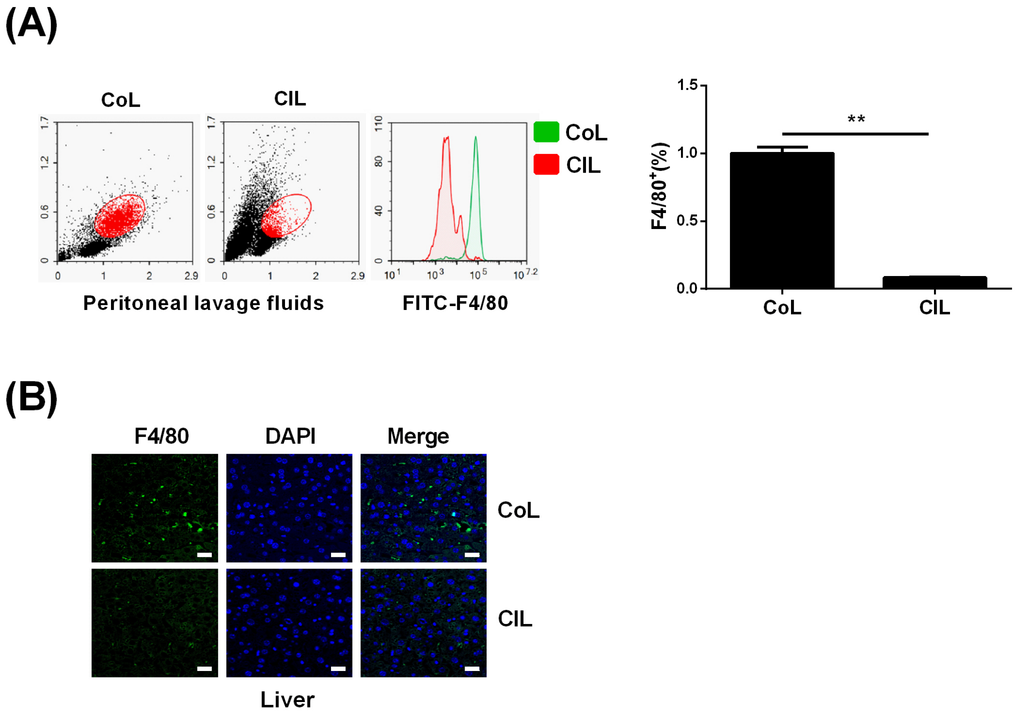

Figure S2. Depletion experiment of macrophages in vivo

(**A-B**) BALB/c mice were injected intravenously daily with either control liposomes (CoL) or clodronate liposomes (ClL) (25 mg/kg body weight each) for 2 consecutive days. Macrophages in peritoneal lavage fluid were stained by FITC-F4/80 and detected (**A**). Liver tissue was stained by FITC-F4/80 and detected (**B**). **: *P*<0.01. *n* = 3. Scale bar = 20 μm.

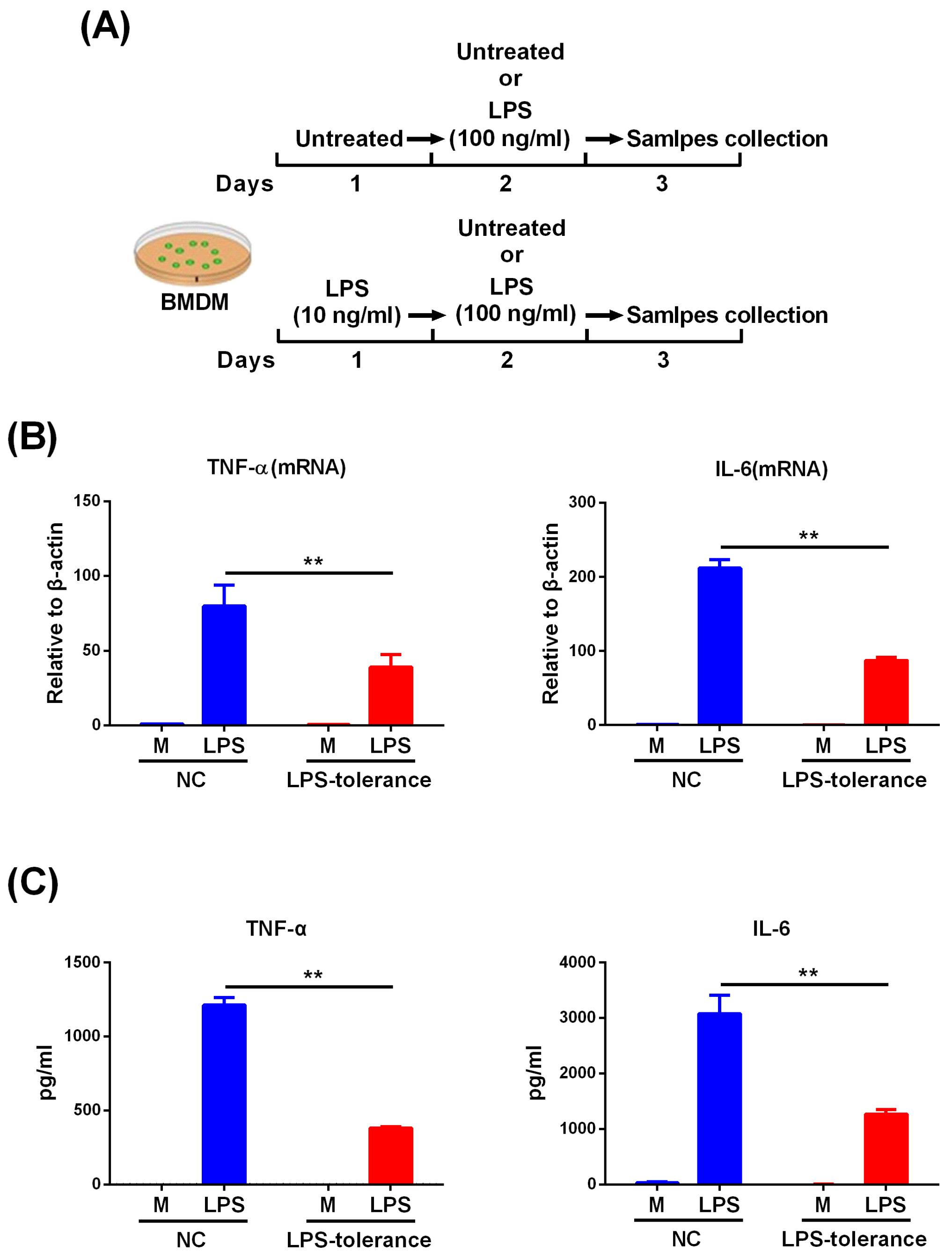

Figure S3. Establishment of LPS tolerance model in macrophage

(**A-C**) BMDMs were untreated (NC) or pretreated with LPS (10 ng/ml) for 24 h, and then untreated or treated with LPS (100 ng/ml) for 24 h (**A**). The mRNA expression of TNF-α and IL-6 (**B**) and the TNF-α and IL-6 of supernatants (**C**) were detected. **: $P<0.01$. $n = 3$.

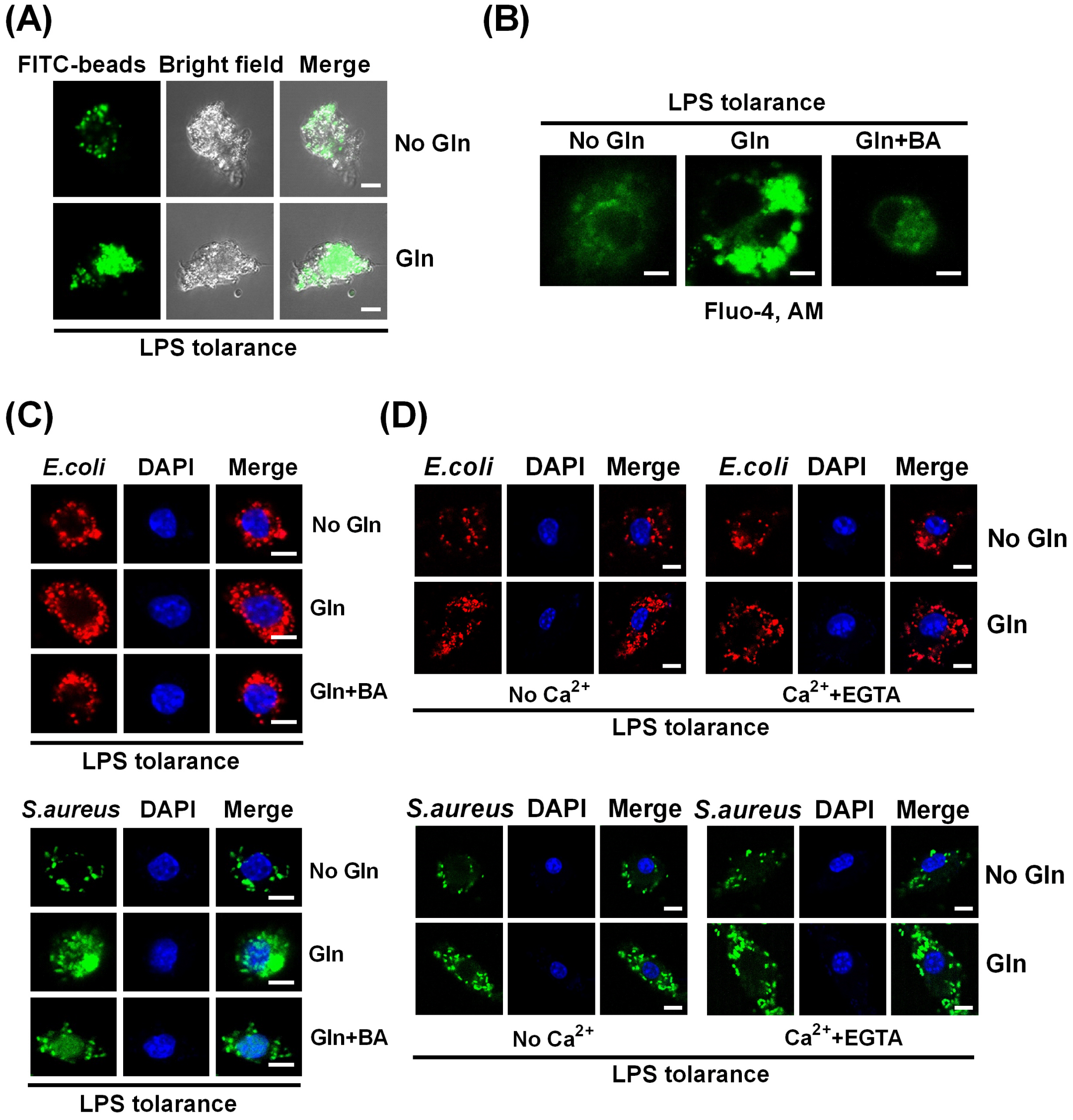

Figure S4. Glutamine promotes macrophage phagocytosis by elevating cytosolic calcium ions

(**A**) LPS-tolerant BMDMs were untreated or treated with glutamine for 24 h. then added fluorescently labeled latex beads (FITC-beads) and incubated for 2 h, Phagocytosis was

analyzed. (**B**) LPS-tolerant BMDMs were incubated in DMEM with or without glutamine, or further treated with BAPTA, AM (BA) for 24 h. Cytosolic calcium ions were detected. (**C**) LPS-tolerant BMDMs were incubated in DMEM with or without glutamine, or further treated with BAPTA, AM for 24 h, and then infected with red-*E. coli* and green-*S. aureus* for 1 h. Phagocytosis was analyzed. (**D**) LPS-tolerant BMDMs were incubated in DMEM containing calcium ions plus EGTA or without calcium ions and further treated with LPS or together with glutamine for 24 h. Cytosolic calcium ions were detected. The concentration of LPS, glutamine and EGTA was 100 ng/ml, 2 mM and 5 mM respectively. The MOI for *E. coli* and *S. aureus* was 1. Scale bar = 5 μm.

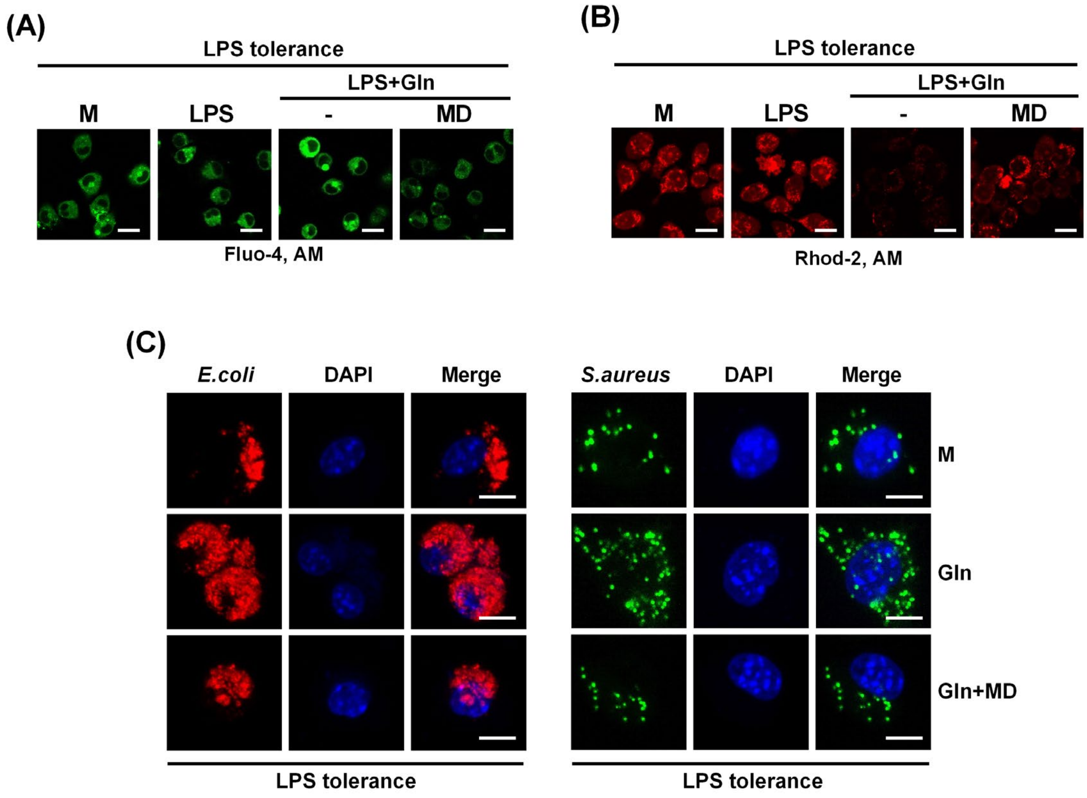

Figure S5. Glutamine elevates cytosolic calcium ions by promoting mitochondrial fission

(**A-B**) LPS-tolerant BMDMs were untreated or treated with LPS alone or together with glutamine or further treated with Mdivi-1 (MD) for 24 h, Fluo-4, AM or Rhod-2, AM was

added and incubated for 30 min. Cytosolic calcium ions (**A**) and mitochondrial calcium ions (**B**) were detected. (**C**) LPS-tolerant BMDMs were incubated in DMEM with or without glutamine, or further treated with Mdivi-1 for 24 h, then infected with *E. coli* and *S. aureus* for 1 h. Phagocytosis was analyzed. The concentration of LPS, glutamine and Mdivi-1 was 100 ng/ml, 2 mM and 10 μM, respectively. The MOI for *E. coli* and *S. aureus* was 1. Scale bar = 10 μm.

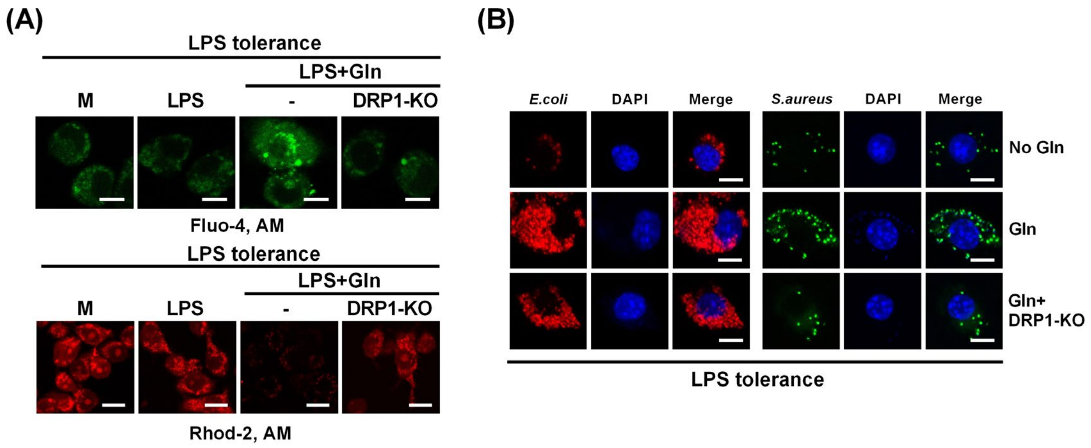

Figure S6. Glutamine promotes cytosolic calcium ion increase, and macrophage phagocytosis mediated by DRP1

(**A**) LPS-tolerant J774A.1 cells or DRP1 knockout (DRP1-KO) LPS-tolerant J774A.1 cells were untreated or treated with LPS or together with glutamine for 24 h, Fluo-4, AM or Rhod-2, AM was added and incubated for 30 min. Cytosolic calcium ions (Scale bar = 5 μm) and mitochondrial calcium ions (Scale bar = 10 μm) were detected. (**B**) LPS-tolerant J774A.1 cells or DRP1 knockout (DRP1-KO) LPS-tolerant J774A.1 cells were incubated in DMEM with or without glutamine for 24 h, then infected with red-*E. coli* and green-*S. aureus* for 1 h. Phagocytosis was analyzed (Scale bar = 5 μm). The concentration of LPS and glutamine was 100 ng/ml, 2 mM, respectively. The MOI for *E. coli* and *S. aureus* was 1.

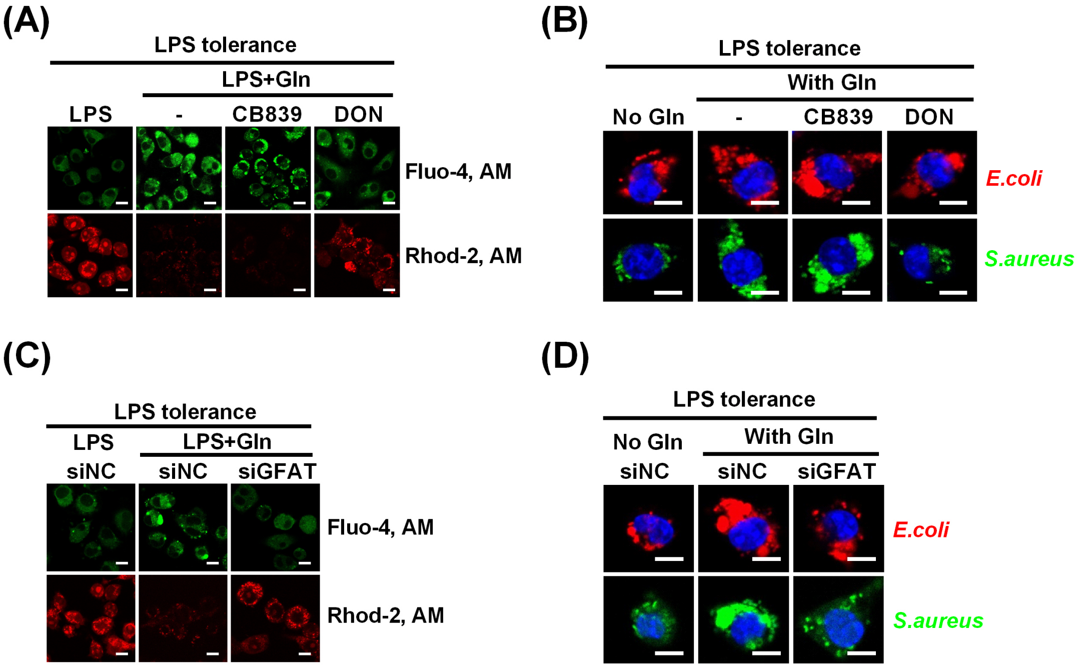

Figure S7. Glutamine promotes cytosolic calcium ion increase, and macrophage phagocytosis mediated by GFAT

(**A**) LPS-tolerant BMDMs were treated with LPS or together with glutamine, or further treated with CB839 or DON for 24 h. Cytosolic calcium ions and mitochondrial calcium ions were detected. (**B**) LPS-tolerant BMDMs were incubated in DMEM with or without glutamine or further treated with CB839 or DON for 24 h. then infected with red-*E. coli* and green-*S. aureus* for 1 h. Phagocytosis was analyzed. (**C**) LPS-tolerant J774A.1 cells were transfected with control siRNA (siNC) or GFAT1 and 2 siRNA (siGFAT) for 48 h and then untreated or treated with LPS alone, or together with glutamine for 24 h. Cytosolic calcium ions and mitochondrial calcium ions were detected. (**D**) LPS-tolerant J774A.1 cells were transfected with control siRNA (siNC) or GFAT1 and 2 siRNA (siGFAT) for 48 h and then untreated or treated with glutamine for 24 h, then infected with red-*E. coli* and green-*S. aureus* for 1 h. Phagocytosis was analyzed. The concentration of LPS, glutamine, CB839 and DON was 100 ng/ml, 2 mM, 10 μM and 50 μM, respectively. The MOI for *E. coli* and *S. aureus* was 1. Scale bar = 10 μm.

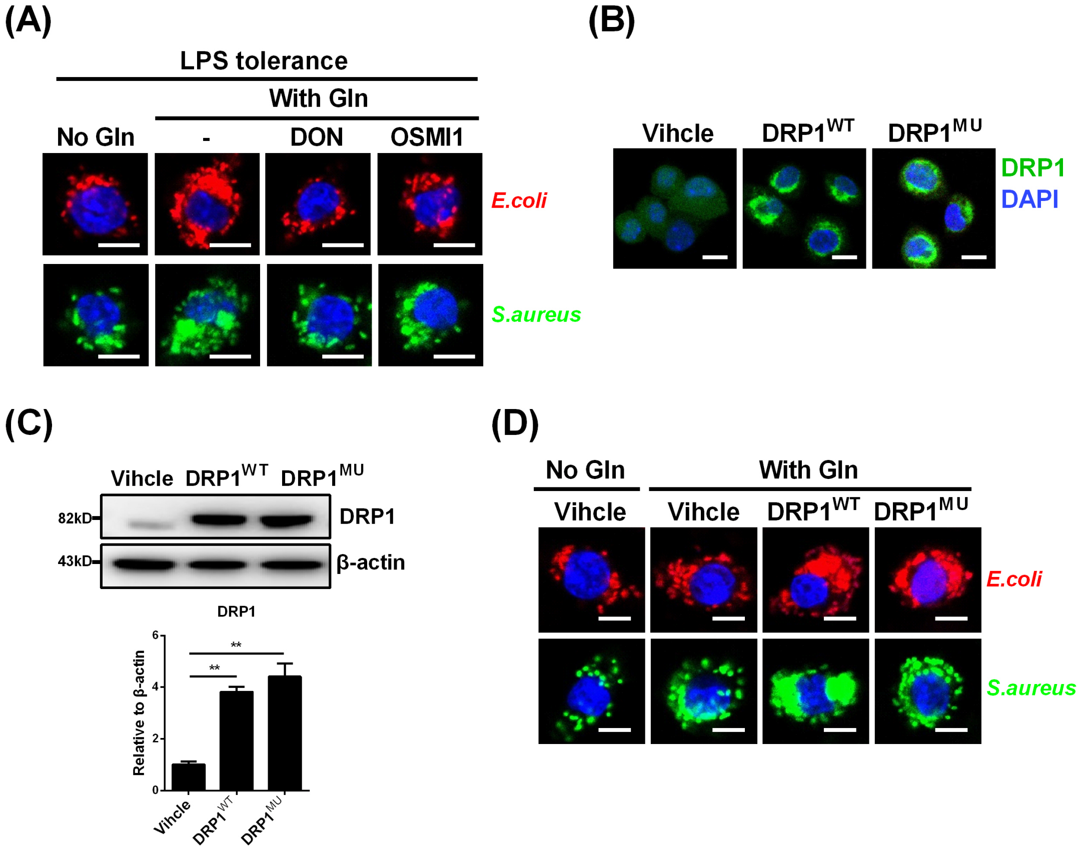

Figure S8. GFAT-mediated DRP1 O-GlcNAcylation promotes macrophage phagocytosis induced by glutamine

(A) LPS-tolerant BMDMs were treated with LPS alone or together with glutamine, or further treated with DON (50 μM) or OSMI1 (10 μM) for 24 h, then infected with red-*E. coli* and green-*S. aureus* for 1 h. Phagocytosis was analyzed. (**B-C**) DRP1-knockout J774A.1 cells were infected with overexpression lentivirus at a multiplicity of infection of 20 for 72 h, then the cells were selected by exposure to puromycin (10 μg/ml) for 24h. Immunofluorescence staining (**B**) and protein expression (**C**) of DRP1 was detected. (**D**) LPS-tolerant DRP1^{WT} and DRP1^{MU} J774A.1 cells were incubated in DMEM with or without glutamine for 24 h, then infected with red-*E. coli* and green-*S. aureus* for 1 h. Phagocytosis was analyzed. The concentration of LPS

and glutamine was 100 ng/ml, 2 mM, respectively. The MOI for *E. coli* and *S. aureus* was 1. **: $P<0.01$. $n = 3$. Scale bar = 10 µm.

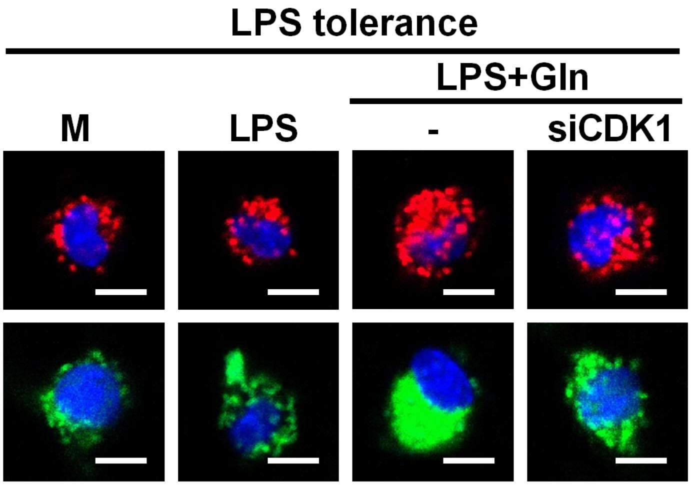

Figure S9. Glutamine promotes macrophage phagocytosis mediated by CDK

LPS-tolerant J774A.1 cells were transfected with control siRNA (siNC) or CDK1 siRNA (siCDK1) for 48 h and then untreated or treated with LPS alone or together with glutamine for 24 h and then infected with red-*E. coli* and green-*S. aureus* for 1 h. Phagocytosis was analyzed. The concentration of LPS and glutamine was 100 ng/ml, 2 mM, respectively. The MOI for *E. coli* and *S. aureus* was 1. **: $P<0.01$. Scale bar = 10 µm.
